# Sequential Acquisition of Virulence and Fluoroquinolone Resistance Has Shaped the Evolution of *Escherichia coli* ST131

**DOI:** 10.1128/mBio.00347-16

**Published:** 2016-04-26

**Authors:** Nouri L. Ben Zakour, Areej S. Alsheikh-Hussain, Melinda M. Ashcroft, Nguyen Thi Khanh Nhu, Leah W. Roberts, Mitchell Stanton-Cook, Mark A. Schembri, Scott A. Beatson

**Affiliations:** a Australian Infectious Diseases Research Centre, School of Chemistry and Molecular Biosciences, The University of Queensland, Brisbane, Australia; b Australian Centre for Ecogenomics, School of Chemistry and Molecular Biosciences, The University of Queensland, Brisbane, Australia

## Abstract

*Escherichia coli* ST131 is the most frequently isolated fluoroquinolone-resistant (FQR) *E. coli* clone worldwide and a major cause of urinary tract and bloodstream infections. Although originally identified through its association with the CTX-M-15 extended-spectrum β-lactamase resistance gene, global genomic epidemiology studies have failed to resolve the geographical and temporal origin of the ST131 ancestor. Here, we developed a framework for the reanalysis of publically available genomes from different countries and used this data set to reconstruct the evolutionary steps that led to the emergence of FQR ST131. Using Bayesian estimation, we show that point mutations in chromosomal genes that confer FQR coincide with the first clinical use of fluoroquinolone in 1986 and illustrate the impact of this pivotal event on the rapid population expansion of ST131 worldwide from an apparent origin in North America. Furthermore, we identify virulence factor acquisition events that predate the development of FQR, suggesting that the gain of virulence-associated genes followed by the tandem development of antibiotic resistance primed the successful global dissemination of ST131.

## IMPORTANCE

*Escherichia coli* sequence type 131 (ST131) is a recently emerged and globally disseminated multidrug-resistant clone frequently associated with human urinary tract and bloodstream infections. In this study, we have used two large publically available genomic data sets to define a number of critical steps in the evolution of this important pathogen. We show that resistance to fluoroquinolones, a class of broad-spectrum antibiotic used extensively in human medicine and veterinary practice, developed in ST131 soon after the introduction of these antibiotics in the United States, most likely in North America. We also mapped the acquisition of several fitness and virulence determinants by ST131 and demonstrate these events occurred prior to the development of fluoroquinolone resistance. Thus, ST131 has emerged by stealth, first acquiring genes associated with an increased capacity to cause human infection, and then gaining a resistance armory that has driven its massive population expansion across the globe.

## INTRODUCTION

*Escherichia coli* sequence type 131 (ST131) is a recently emerged multidrug-resistant clone associated with urinary tract and bloodstream infections. *E. coli* ST131 was originally identified due to its strong association with the CTX-M-15-type extended-spectrum-β-lactamase (ESBL) allele (1), and is now the predominant fluoroquinolone-resistant (FQR) *E. coli* clone worldwide (2–4).

ST131 belongs to subgroup 1 from *E. coli* phylogroup B2, with most strains of serotype O25b:H4 (1–4). Two previous genomic studies have explored the ST131 clonal structure (2, 5) and identified a globally dominant FQR sublineage defined as clade C (2), or *H*30-R (5). Two additional well-supported ST131 sublineages, referred to as clades A and B, have also been described (2). Each of these clades contains a defined marker allele for the type 1 fimbriae *fimH* adhesin: *H*41 in clade A, *H*22 in clade B, and *H*30 in clade C (6). Further analysis of clade C/*H*30-R ST131 identified a smaller subset of strains containing the *bla*_CTX-M-15_ ESBL allele referred to as clade C2, or *H*30-Rx (2, 5). The ST131 strain EC958 is a reference FQR clade C strain that has been well characterized at the genomic and phenotypic level (3, 7–12).

Several early studies demonstrated variation in the complement of virulence genes in ST131, with only a few virulence factors consistently identified in all strains (1, 4, 13–15). Our comprehensive analysis of 95 *E. coli* ST131 genomes revealed that the virulence and mobile genetic element (MGE) profile was in fact consistent with the phylogenetic structure of the ST131 lineage, with clade C strains sharing a generally conserved set of genes. In contrast, the plasmid profile of ST131 is highly disparate, with multiple different replicons found in closely related strains and multiple genomic contexts for the clade C2-defining *bla*_CTX-M-15_ ESBL gene (16, 17).

Despite its successful dissemination globally, little information is available about evolution and emergence of ST131. Two recent independent genomic studies demonstrated that ST131 emerged from a single ancestor and that most strains belong to clade C/*H*30-R (2, 5). Notably, we found that recombination accounted for the majority of variation within the ST131 lineage, and recombination events were associated with the positions of MGEs (2). However, despite the number of isolates in both studies, neither resolved the geographical or temporal origin of the ST131 ancestor. In contrast, studies of other large sets of bacteria with geographical or temporal separation have determined accurate dates of divergence of important clades using statistical analyses such as the Bayesian framework implemented in BEAST (Bayesian Evolutionary Analysis by Sampling Trees) (18). For example, Glaser identified tetracycline resistance as the major driver of diversification among the global population of group B streptococci (19). Similarly, a large study of methicillin-resistant *Staphylococcus aureus* (MRSA) was able to date the emergence of an FQR clade to the mid-1980s (20). These studies motivated us to combine data sets from our geographically diverse previous study (2) and from the temporally diverse study by Price et al. (5) to investigate the evolution of ST131 with the highest possible resolution.

## RESULTS AND DISCUSSION

### Curation of a high-quality ST131 genome sequence data set.

We first sought to obtain a high-quality set of data to carry out our analyses. A total of 199 draft Illumina paired-end *E. coli* ST131 genomes were retrieved from public read data repositories (see Data Set S1 in the supplemental material). Initial phylogenetic analyses of *de novo* and reference-guided assemblies of all 199 *E. coli* ST131 genomes indicated that several draft genomes were of low quality. Suboptimal genome data quality could interfere with subsequent phylogenetic analyses and may invalidate conclusions drawn from tree topologies. To ensure that only high-quality sequences were included in our analyses, we removed 14 genome data sets that were determined to be outliers according to at least one of our assembly or mapping metrics, including number of uncalled bases, number of scaffolds, and assembled genome size (see Fig. S1 in the supplemental material). This quality control (QC) filter is broadly applicable to reanalyzing public genomic data from multiple sources.

### Phylogenomic analysis of ST131.

**FIG 1:**
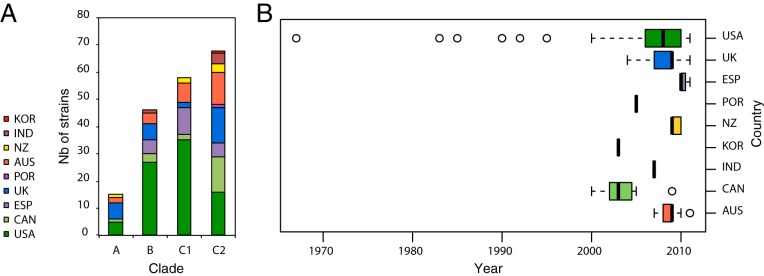
Geographical diversity of the combined data set across clades and time. (A) Stacked histogram showing the number of strains in clades A, B, C1, and C2 according to their country of origin. The color scheme is shown in the legend on the left along with abbreviated country names. (B) Box-and-whisker plot showing the distribution according to year of isolation for all strains based on their country of origin. Country abbreviations: KOR, South Korea; IND, India; NZ, New Zealand; AUS, Australia; POR, Portugal; UK, United Kingdom; ESP, Spain; CAN, Canada; USA, United States of America.

We next carried out phylogenetic reconstruction using our combined data set of 185 Illumina paired-end sequences, which represented strains from humans (*n* = 167), animals (*n* = 15), and other sources (*n* = 3) isolated from the United States, Canada, New Zealand, Australia, Spain, India, Portugal, Korea, and the United Kingdom between 1967 and 2011 (Fig. 1; see Data Set S1 in the supplemental material). Sequence read mapping of these 185 high-quality ST131 genomes (and simulated reads from the SE15, EC958, and JJ1886 complete genomes) to the ST131 clade C reference genome *E. coli* EC958 defined 21,373 substitution single nucleotide polymorphisms (SNPs) that were used to create an unrooted maximum likelihood (ML) phylogeny (see Fig. S2A in the supplemental material). An independent phylogenetic tree produced by the kSNP alignment-free method was consistent with the overall topology of the ML trees (see Fig. S3 in the supplemental material). Using a Bayesian modeling algorithm, we identified 204 nonoverlapping segments encompassing 1.542 Mb and containing 15,902 substitution SNPs that were introduced into the ST131 lineage by recombination (see Fig. S4 and Data Set S1 in the supplemental material). The length of recombinant sequence is higher than previously reported (2) as the larger data set increases the probability that one strain will have a recombinant fragment not encountered before. The length of the nonrecombinant core ST131 genome is 0.19 Mb less than previously reported (2), encompassing 69.4% of the EC958 chromosome, or approximately 3.55 Mb. However, the proportion of SNPs introduced by recombination (74.4%) is consistent with our previous study and highlights the important role recombination has played in shaping the ST131 lineage (2). Exclusion of these recombinant SNPs from phylogenetic analyses reduced the number of SNPs to 5,471 and resulted in a tree that maintained the original overall topology, albeit with substantially reduced branch lengths and some major within-clade reclustering of strains (see Fig. S2B). Consistent results were achieved using an independent method of recombination detection and removal indicating that our tree topologies are not biased by the chosen methodology (see Fig. S5 in the supplemental material).

**FIG 2:**
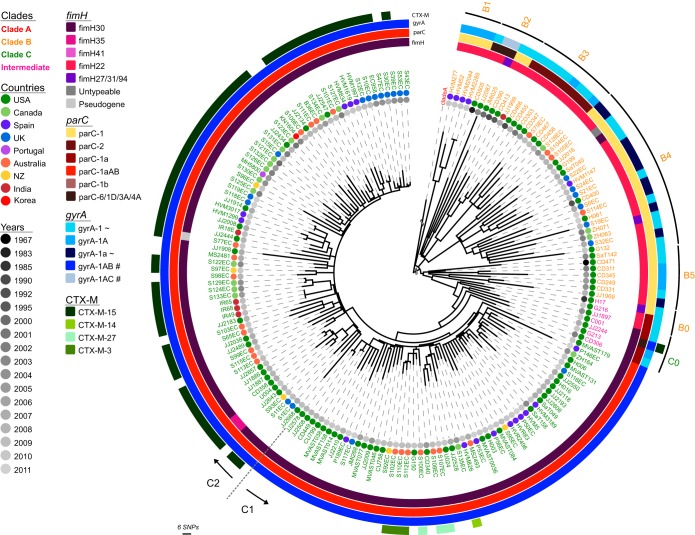
Maximum likelihood phylogenetic tree of ST131 strains. The phylogram was built from 5,471 nonrecombinant SNPs using maximum likelihood (ML). Branch support was performed by 1,000 bootstrap replicates (see Fig. 2B in the supplemental material). The scale bar indicates the number of substitution SNPs. Taxon labels for clades A, B, and C are colored red, orange, and green, respectively. Seven strains sharing intermediate characteristics between clades B and C are colored pink. Of note, clade A strains were collapsed and the clade A-specific branches shortened for display. Metadata are represented as circles as follows: year of isolation in gray and gradient and geographical region in assorted colors as depicted in the legend. Allelic profiling information is shown as colored strips surrounding the phylogram (from inner to outer) for the *fimH*, *parC*, *gyrA*, and CTX-M genes. Two additional distinctions were made for some *fimH* variants: “Untypeable” corresponds to a strain with a truncated or missing *fimH* gene, and “Pseudogene” corresponds to a strain in which *fimH* is disrupted by an insertion sequence. Clades B0 to B5 and C0 subclades are shown as arcs in the outermost ring, with arrows and dotted lines denoting the division between subclades C1 and C2.

**FIG 3:**
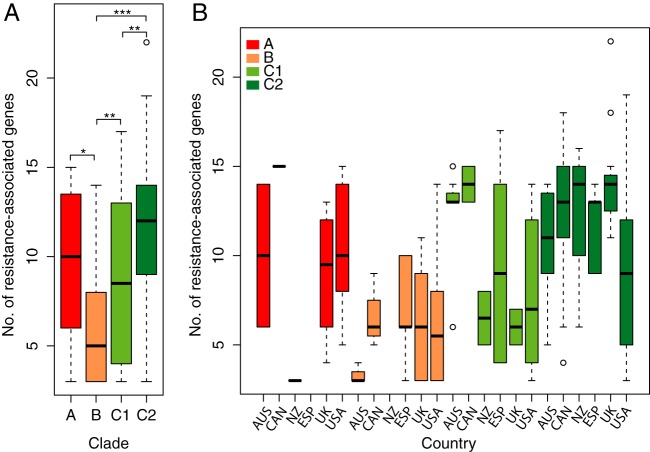
Prevalence of antibiotic resistance-associated genes by (A) clade alone and (B) clade and country. The box-and-whisker plot shows the number of resistance-associated genes per (A) clade and (B) per clade and country. Colors correspond to clades as follows: clade A, red; clade B, orange; clade C1, light green; and clade C2, dark green. Screening was done using Srst2 (42) against ARGannot, with a minimum of depth of 15× read coverage. No., number. Country abbreviations: AUS, Australia; CAN, Canada; NZ, New Zealand; ESP, Spain; USA, United States of America; UK, United Kingdom. *P* values are indicated as follows: ***, *P* < 0.001; **, *P* < 0.01; and *, *P* < 0.05.

Extending our phylogenomic analyses to include isolates from two large international collections provided a far greater resolution of the evolution within the ST131 lineage (Fig. 2). The global phylogeny of *E. coli* ST131 separated the strains into three distinct lineages (clades A, B, and C). Congruent with our previous work, strains in clade C were characterized by the *fimH30* allele and the FQR-conferring alleles *gyrA1AB* and *parC1aAB* (Fig. 2). Notable exceptions were strains JJ2643 and U004 in clade C, which contain the *fimH35* allele. This appeared to be due to a recombination event encompassing *fimH* in these strains and highlights why we have retained a neutral nomenclature (i.e., A, B, and C) for our clade classifications. Likewise, the CTX-M-15 allele is not ubiquitous in all clade C2 strains, making this a more scalable classification than the *H*30-Rx designation originally suggested by Price et al. (5) (Fig. 2). In addition to harboring CTX-M-15 genes, clade C2 strains contain more resistance genes in total compared with other ST131 clades (Fig. 3), consistent with colocalization of multiple plasmid-encoded resistance genes (see Data Set S1 in the supplemental material). Although the context of multidrug resistance cassettes can be resolved in some cases from draft genome data from ST131 isolates or transformants (16, 17), the full complexity of plasmid-mediated resistance in ST131 requires the generation of more complete genomes as per EC958 and JJ1886 (7, 21).

### A combined data set enables greater resolution of ST131 subclades.

**FIG 4:**
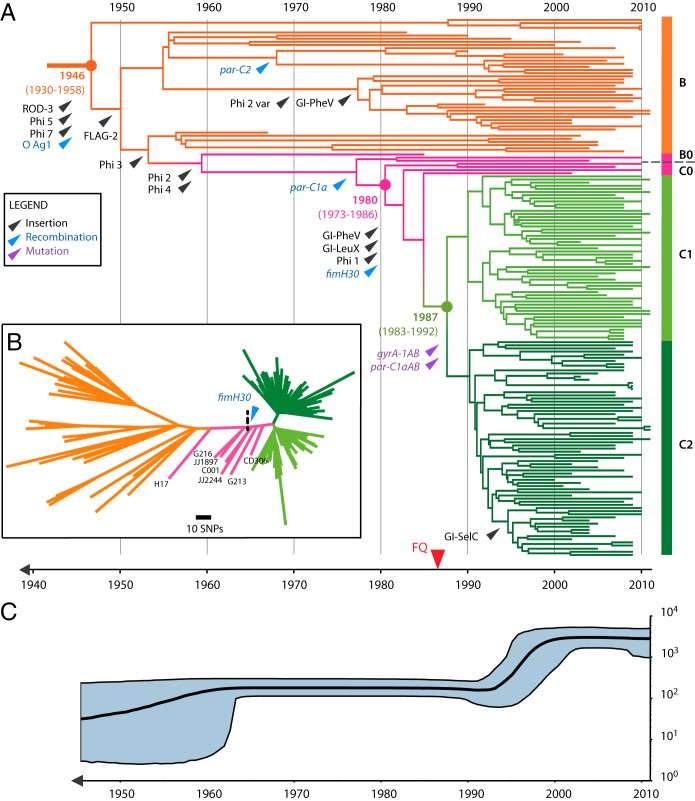
Evolutionary scenario of the emergence of ST131 clades (B and C) A time-calibrated phylogeny was reconstructed using BEAST 2.0 based on 3,779-bp nonrecombinant SNPs for the 172 clade B and C strains. Of all combinations tested (see the summary of BEAST analysis results in Data Set S1 in the supplemental material), the one combining the GTR substitution model, a constant relaxed clock model, and the Bayesian skyline population tree model was preferred. (A) Maximum clade credibility tree colored according to clade origin as shown on the right with B in orange, intermediate B0 and C0 in pink, C1 in light green, and C2 in dark green. The *x* axis indicates emergence time estimates of the corresponding strains. Major evolutionary events are also indicated by an arrow pointing at the branch onto which they are predicted to have occurred (the position along the branch is arbitrary). Three categories of major events are displayed, namely, MGE and genomic island (GI) insertion events in black, allelic change acquired through recombination events in blue, and allelic change acquired through point mutation events in purple. Of note, the two point mutations indicated by purple arrowheads and pointing at the branch from which clades C1 and C2 originate confer resistance to fluoroquinolone, for which the first introduction is indicated in the bottom timeline by a red arrowhead. (B) Unrooted phylogenetic tree built on the same 3,779-bp nonrecombinant set of SNPs using maximum likelihood (ML). Branch support was performed by 1,000 bootstrap replicates. Intermediate strain names and predicted acquisition of *fimH30* are indicated on the tree. (C) The Bayesian skyline plot illustrates the predicted demographic changes of the ST131 clade B and C population since the mid-1940s. The black curve indicates the effective population size (Ne), with 95% confidence intervals shown in blue.

The combined ST131 data set enabled greater resolution of the differences between clade B and C strains. We previously showed that clade C strains can be further segregated into two distinct subclades, C1 and C2 (2). Our new analysis defined five discrete subclades in clade B (B1 to B5), each with distinct repertoires of selected marker genes *fimH*, *parC*, and *gyrA* (Fig. 2). Intriguingly, we found that seven strains originating from the United States could be classified as “intermediate” on the basis of their SNP pattern (Tables 1 and 2). These strains showed progressive acquisition of clade C-defining point mutations, with the three isolates closer to clade B (classified B0) and four isolates closer to clade C (classified C0) illustrating the precise evolutionary events leading to the emergence of clade C (Fig. 4).

**TABLE 1:** Clade-specific SNPs identified between clades B and C

SNP position in EC958	SNP identified in clade^a^:									Impact^b^	Locus tag	Codon	Gene	Product
	B	B0			C0				C					
		H17	G216	JJ1897	C001	JJ2244	G213	CD306						
1244787	**C**	**C**	**C**	**C**	**C**	**C**	**C**	**C**	*T*	I→V	EC958_1322	23	*potB*	Spermidine-putrescine transport system permease
2715324	**G**	**G**	**G**	**G**	**G**	**G**	**G**	**G**	*A*	Syn	EC958_5020	125		Hypothetical protein
3536312	**C**	**C**	**C**	**C**	**C**	**C**	**C**	**C**	*T*					
3555316	**C**	**C**	**C**	**C**	**C**	**C**	**C**	**C**	*T*	Syn	EC958_3511	39	*yhaK*	Hypothetical protein
3921426	**G**	**G**	**G**	**G**	**G**	**G**	**G**	**G**	*A*	K→R	EC958_3887	78	*yhiM*	Hypothetical protein
2536291	**C**	**C**	**C**	**C**	**C**	*T*	**C**	**C**	*T*	N→D	EC958_2567	87	*gyrA*	DNA gyrase subunit A
1504736	**C**	**C**	**C**	**C**	**C**	**C**	**C**	*G*	*G*	Syn	EC958_1601	142	*yciK*	Hypothetical oxidoreductase YciK
2744500	**C**	**C**	**C**	**C**	**C**	**C**	**C**	*T*	*T*	I→V	EC958_2766	224	*eutB*	Probable regulatory subunit of ethanolamine ammonia-lyase
2937986	**A**	**A**	**A**	**A**	**A**	**A**	**A**	*G*	*G*					
4032999	**C**	**C**	**C**	**C**	**C**	**C**	**C**	*T*	*T*	C→R	EC958_3982	119	*yiaK*	Hypothetical oxidoreductase YiaK
1382365	**G**	**G**	**G**	**G**	**G**	**G**	*A*	*A*	*A*	Syn	EC958_1478	204	*hemA*	Glutamyl-tRNA reductase
2756734	**C**	**C**	**C**	**C**	**C**	**C**	*T*	*T*	*T*	Syn	EC958_2779	448	*maeB*	NADP-dependent malate dehydrogenase
3883417	**G**	**G**	**G**	**G**	**G**	**G**	*A*	*A*	*A*	M→T	EC958_3846	231	*livG*	ATP-binding component of high-affinity branched-chain amino acid transport system LivG
53758	**T**	**T**	**T**	**T**	*A*	*A*	*A*	*A*	*A*	R→S	EC958_0181	147	*kefC*	Glutathione-regulated potassium efflux antiporter
2705332	**G**	**G**	**G**	**G**	*A*	*A*	*A*	*A*	*A*	Syn	EC958_5013	334	*nupC*	Nucleoside-transport system protein NupC
3549826	**A**	**A**	**A**	**A**	*T*	*T*	*T*	*T*	*T*	F→Y	EC958_3501	184	*yqjA*	Hypothetical protein
3587935	**G**	**G**	**G**	**G**	*A*	*A*	*A*	*A*	*A*	N→D	EC958_3544	20	*agaD*	Phosphotransferase system, *N*-acetylgalactosamine-specific IID component
642980	**C**	**C**	*T*	*T*	*T*	*T*	*T*	*T*	*T*	Syn	EC958_0717	116	*ybdB*	Hypothetical protein
761921	**G**	**G**	*A*	*A*	*A*	*A*	*A*	*A*	*A*	Syn	EC958_0832	150		Putative C_4_-dicarboxylate transporter, small permease protein (DctQ-like)
1001668	**T**	**T**	*C*	*C*	*C*	*C*	*C*	*C*	*C*	Syn	EC958_1062	115	*ycaP*	Hypothetical protein
1167464	**C**	**C**	*T*	*T*	*T*	*T*	*T*	*T*	*T*	C→R	EC958_1238	268	*ymdC*	Putative synthase
1317794	**G**	**G**	*A*	*A*	*A*	*A*	*A*	*A*	*A*	Syn	EC958_1416	36	*ycgK*	Protein YcgK
1727956	**G**	**G**	*T*	*T*	*T*	*T*	*T*	*T*	*T*	S→A	EC958_1806	223		Probable dimethyl sulfoxide reductase chain YnfF
2191491	**C**	**C**	*T*	*T*	*T*	*T*	*T*	*T*	*T*	Syn	EC958_2289	527		Putative peptide synthetase-like protein
2395814	**C**	**C**	*T*	*T*	*T*	*T*	*T*	*T*	*T*	L→P	EC958_2450	556		Hypothetical protein
2686010	**T**	**T**	*C*	*C*	*C*	*C*	*C*	*C*	*C*					
2692404	**C**	**C**	*T*	*T*	*T*	*T*	*T*	*T*	*T*	C→R	EC958_5002	238	*ypdB*	Hypothetical protein
2706426	**C**	**C**	*T*	*T*	*T*	*T*	*T*	*T*	*T*	Syn	EC958_5222	482	*yfeA*	Hypothetical protein
3030230	**G**	**G**	*T*	*T*	*T*	*T*	*T*	*T*	*T*	K→N	EC958_3037	192		Hypothetical protein
3798899	**G**	**G**	*A*	*A*	*A*	*A*	*A*	*A*	*A*	Syn	EC958_3771	208	*yhfW*	Hypothetical protein
3802091	**G**	**G**	*T*	*T*	*T*	*T*	*T*	*T*	*T*					
3823737	**A**	**A**	*G*	*G*	*G*	*G*	*G*	*G*	*G*					
3913371	**G**	**G**	*A*	*A*	*A*	*A*	*A*	*A*	*A*	T→A	EC958_3880	195		Putative xylulose kinase
4009795	**C**	**C**	*T*	*T*	*T*	*T*	*T*	*T*	*T*	V→A	EC958_3959	327	*yiaE*	Putative dehydrogenase
4216222	**C**	**C**	*T*	*T*	*T*	*T*	*T*	*T*	*T*					
4233771	**T**	**T**	*C*	*C*	*C*	*C*	*C*	*C*	*C*					
779389	**C**	*T*	*T*	*T*	*T*	*T*	*T*	*T*	*T*	L→P	EC958_0846	476		Putative glycosyl hydrolase
822709	**G**	*A*	*A*	*A*	*A*	*A*	*A*	*A*	*A*	Syn	EC958_0888	281	*bioF*	8-Amino-7-oxononanoate synthase
2406295	**G**	*A*	*A*	*A*	*A*	*A*	*A*	*A*	*A*					
3892567	**A**	*G*	*G*	*G*	*G*	*G*	*G*	*G*	*G*	S→F	EC958_3856	69	*ftsX*	Cell division membrane protein
3941887	**G**	*A*	*A*	*A*	*A*	*A*	*A*	*A*	*A*	Syn	EC958_3906	72	*chuW*	Putative oxygen-independent coproporphyrinogen III oxidase
4631378	**C**	*T*	*T*	*T*	*T*	*T*	*T*	*T*	*T*	K→E	EC958_4497	567	*plsB*	Glycerol-3-phosphate acyltransferase

a Boldface indicates clade B specific, and italic indicates clade C specific.

b Impact of SNP in bold relative to ST131 clade C2 strain EC958. Syn, synonymous change; non-synonymous changes to protein-coding genes are shown with single letter amino acid codes (EC958 sequence on left, SNP impact on right); blank lines indicate SNP in intergenic region.

**TABLE 2:** Clade-specific SNPs identified between clades C1 and C2

SNP position in EC958	SNP in clade^a^:						Impact^b^	Locus tag	Codon	Gene	Product
	C0				C1	C2					
	C001	JJ2244	G213	CD306							
426936	**C**	**C**	**C**	**C**	**C**	*T*	V→A	EC958_0513	295	*sbmA*	Peptide antibiotic transporter SbmA
737849	**G**	**G**	**G**	**G**	**G**	*A*					
2838072	**G**	**G**	**G**	**G**	**G**	*A*	Syn	EC958_2841	190	*iscS*	Cysteine desulfurase
2878095	**C**	**C**	**C**	**C**	**C**	*T*	E→G	EC958_2875	229	*lepA*	GTP-binding elongation factor LepA
3710995	**C**	**C**	**C**	**C**	**C**	*T*	Syn	EC958_4822	302	*acrF*	Acriflavine resistance protein F
3905668	**C**	**C**	**C**	**C**	**C**	*T*	S→P	EC958_3870	451	*nikA*	Nickel-binding periplasmic protein
4100042	*C*	*C*	*C*	*C*	**T**	*C*					
4397873	*G*	*G*	*G*	*G*	**A**	*G*	S→N	EC958_4314	395	*rmuC*	DNA recombination protein RmuC

a Boldface indicates clade C1 specific, and italic indicates clade C2 specific.

b Impact of SNP in bold relative to ST131 clade C2 strain EC958. Syn, synonymous change; non-synonymous changes to protein-coding genes are shown by single letter amino acid codes (EC958 sequence on left, SNP impact on right); blank lines indicate SNP in intergenic region.

Strains from the five clade B sublineages varied in their *parC* and *gyrA* allelic profile, with the vast majority of clade B strains containing allele combinations that are not associated with fluoroquinolone resistance. Additionally, while all strains from subclades B2 and B5 are associated with the United States, and B1 with Spain, strains within subclades B3 and B4 have a more diverse geographic origin. Each subclade showed a distinctive recombination profile (see Fig. S4 and S5 in the supplemental material) and MGE repertoire (see Fig. S6 in the supplemental material), indicative of independent evolutionary trajectories. In contrast, we found that the prevalence of virulence genes is largely conserved across all B subclades, with the absence of several uropathogenic *E. coli* (UPEC)-specific genes apparent in clade B3 (see Fig. S7 in the supplemental material). By comparison, our investigation of clade C MGEs and other regions of interest (as originally defined in the clade C reference strain EC958) showed a high degree of conservation across clade C, with the exception of the prophage Phi6, the capsule loci, and genomic island GI-*selC* (see Fig. S8 in the supplemental material). For example, GI-*selC* is only found in a geographically homogeneous cluster of clade C strains that include EC958 and excludes the reference strain JJ1886 (Fig. 2; see Fig. S6). Despite the general conservation of gene content within clade C genomes, it is apparent that genomic islands are hot spots for insertions, deletions (indels), and genome rearrangement (see Fig. S6 and Data Set S1 in the supplemental material). EC958 GI-*pheV* has several small indels relative to JJ1886 GI-*pheV*, and we have previously shown that the CMY-23 β-lactamase gene that confers resistance to third-generation cephalosporins has inserted within the EC958 GI-*leuX*, whereas the JJ1886 GI-*leuX* element has a large duplication relative to EC958 (12) (see Data Set S1).

### Temporal analysis of ST131 identifies major divergence dates.

Our initial studies of ST131 strains collected between 2001 and 2011 showed insufficient temporal depth to robustly date the emergence of clade C (2). By including 91 more strains from Price et al. (5), including 8 that predated 2000 (Fig. 1), we anticipated that we would be able to resolve this question using existing public data alone. We generated a linear regression of the genetic distance from the root to tip against time for the 172 ST131 isolates within clades B and C using Path-O-Gen (22). This analysis revealed a positive correlation (*R*^2^ = 0.3233, *P* < 0.0001) confirming the molecular clock-like signal (see Fig. S8 in the supplemental material). To accurately estimate the date of divergence of clade C from clade B we employed BEAST (18). BEAST analysis rejected the strict clock and favored the uncorrelated log-normal clock model in combination with a Bayesian skyline population model (see Data Set S1 in the supplemental material). A mutation rate of 4.39 × 10^−7^ SNPs per site per year (95% highest posterior density [HPD], 3.58 × 10^−7^ to 5.23 × 10^−7^) was calculated, consistent with other large-scale phylogenomic analyses of the *E. coli*/*Shigella* lineage (6.0 × 10^−7^ SNPs per site per year [95% HPD, 5.2 × 10^−7^ to 6.7 × 10^−7^]) (23) (see Fig. S9 in the supplemental material). Based on this approach, we could estimate the divergence of the last common ancestor of clade B and C strains to have occurred between 1930 and 1958 (Fig. 4A), consistent with the Path-O-Gen prediction (see Fig. S8a). We could estimate the divergence of clade C from clade B to have occurred in 1980 (95% HPD, 1973 to 1986), which was slightly earlier than the Path-O-Gen prediction (see Fig. S8a). Importantly, we identified that further diversification of clade C2 from clade C1 dated to 1987 (95% HPD, 1983 to 1992), subsequent to all clade C1 and C2 strains acquiring *gyrA1AB* and *parC1aAB* alleles imparting elevated FQR (Fig. 4A and 4B). Bayesian skyline plots show a relatively constant population size over several decades, followed by a short recent expansion occurring in the late 1990s and early 2000s and subsequent stabilization (Fig. 4C). Interestingly, this pattern is consistent with the introduction of FQ for clinical use in 1986 (24) and the subsequent stabilization may reflect the improved stewardship of FQ (or its removal from general use). A similar phenomenon was observed for FQR among the members of the MRSA ST22 global phylogeny (20). Remarkably, an identical date was identified in a recent preprint report using 81 ST131 genomes from Price et al. (5), supplemented with ~100 newly sequenced ST131 genomes from more geographically dispersed strains (17), highlighting the value of careful analysis of existing data sets. Although acquisition of the CTX-M-15 gene within clade C2 may be a major factor in the diversification of C1 and C2, it is worth emphasizing that this alone does not explain the success of ST131 given that the population expansion identified in our study encompasses both clade C1 and clade C2 strains.

### Phylogeography of ST131.

**FIG 5:**
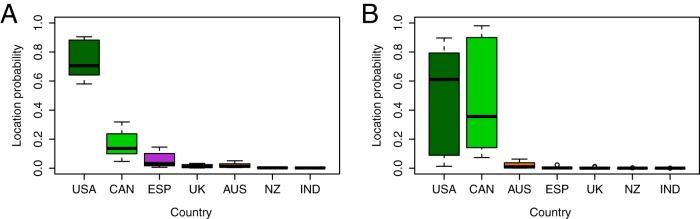
Geographic location of the most recent common ancestor (MRCA) of ST131 major clades. Individual probabilities were predicted from 10 independent BEAST analyses of randomly subsampled data to limit bias related to overrepresentation of some locations. The mean probabilities of the geographic locations of the MRCA for (A) clades B and C and (B) clade C are shown as a box-and-whiskers plot colored according to country using the scheme as described in Fig. 1. Countries are labeled on the *x* axis by abbreviation: USA, United States of America; CAN, Canada; ESP, Spain; UK, United Kingdom; AUS, Australia; NZ, New Zealand; IND, India.

To investigate the geographical context underlining the expansion of the multidrug-resistant ST131 O25b:H4 clone from clades B to C, we performed a discrete phylogeographical analysis as implemented in BEAST on the 172 ST131 isolates within clades B and C that included a variety of geographic sources (Fig. 1A) and dates (Fig. 1B). To reduce sampling biases due to the high number of strains isolated from United States, Canada, United Kingdom, and Spain, we performed independent analyses on 10 randomly subsampled data sets containing 85 strains each (see Data Set S1 in the supplemental material). Under Bayesian stochastic search variable selection (BSSVS) and symmetric diffusion models, results systematically supported the United States (74.31%; standard deviation [SD], 12.1%) as the most likely origin of clades B and C (Fig. 5A). The origin of clade C (C0, C1, and C2) was predicted to be associated with either the United States (51.83%; SD, 35.5%) or Canada (45.59%; SD, 36.1%), over all of North America (Fig. 5B). These results are consistent with the observation that the oldest reported ST131 strain was isolated in the United States in 1967 (5), as well as an independent BEAST analysis using a partly overlapping data set (17). Although our resampling approach has minimized bias in strain origin, a data set with greater diversity of strains from different geographical regions and pre-2000 isolation dates would be necessary to rule out a different origin (e.g., current data sets are underrepresented in South America, Africa, and many European and Asian countries). A greater number of strains would also help identify local outbreaks or clusters: with the exception of GI-*selC* carrying clade C strains from the United Kingdom, which cluster phylogenetically (Fig. 2), we did not observe other significant geographic clustering using this data set alone.

### Intermediate strains reveal key MGE acquisitions that define clade C.

Overall, excluding intermediate B0 and C0 strains, clade C differs from clade B by only 42 substitution SNPs (Tables 1 and 2). This list included the majority of the 70 clade C-defining SNPs reported in our earlier study (2), but was not identical due to the greater number of recombinant regions identified and removed in the present study. Closer examination of the recombination analysis identified intermediate patterns of recombination, primarily clustered around known MGEs, indicative of stepwise evolution among these intermediate strains (see Fig. S4 and S5 in the supplemental material). Most notably, we could trace the acquisition of GI-*pheV* and GI-*leuX* genomic islands to the most recent common ancestor of the C0 strains (C001, JJ2244, G213, and CD306), several years before the acquisition of the FQR mutations that define clade C (Fig. 4). The *pheV* genomic island acquired by clade C ST131 strains is known to carry the autotransporter genes *agn-43* and *sat*, the ferric aerobactin biosynthesis gene cluster (*iucABCD*), and its cognate ferrisiderophore receptor gene *iutA* (3, 7). The clade-defining *fimH30* allele was acquired by recombination (25), possibly in conjunction with the acquisition of the nearby GI*-leuX* island; the same time point is also predicted for the acquisition of the IS*Ec55* insertion sequence within the *fimB* recombinase gene that we have previously shown to affect the expression of type 1 fimbriae (3, 7). Thus, close scrutiny of these “intermediate” genomes enabled us to trace the acquisition of virulence-associated genes in ST131, which appears to have primed this clone for success prior to the acquisition of FQR mutations in the late 1980s. Further molecular analysis is required to determine the contribution of these elements to ST131 colonization/fitness in the gastrointestinal and urinary tracts. Notably, the role of virulence in the success of this clone may have been underappreciated in a recent report as these particular strains were distributed throughout clades B and C despite their inconsistent *parC*, *gyrA*, and *fimH* alleles (17). The B0 and C0 strains were also dispersed in our tree that includes recombinant regions (see Fig. S2A in the supplemental material), suggesting that differences in recombination removal may account for these discrepancies. However, we cannot rule out differences in phylogeny due to the particular set of genomes analyzed. Future studies combining more publicly available genome datasets will help to further reveal the precise evolutionary trajectory taken by the globally disseminated ST131 clone.

### Conclusions.

Overall, our work highlights how careful reanalysis of publicly available genomic data sets from heterogeneous sources can greatly improve the resolution of evolutionary history. Here, we have characterized the evolution of ST131 with unprecedented detail, from the acquisition of prophages and modification of the O antigen region ca. 1946, to the acquisition of GI-*pheV*, GI-*leuX*, *fimH30*, and IS*Ec55* around 1980, several years before the acquisition of mutations in *gyrA* and *parC* that led to FQR and the acquisition of the clade C2-defining CTX-M-15 ESBL gene. Whereas the development of FQR was accompanied by a large surge in the ST131 population globally, we propose that the acquisition of virulence factors by ST131 was a necessary precursor to this success. These events describe the “perfect storm” for the evolution of a multidrug-resistant pathogen: the acquisition of virulence-associated genes followed by the development of antibiotic resistance.

## MATERIALS AND METHODS

### Genome data.

Two *E. coli* ST131 strain data sets from previously published work were used in this study, under the designations data set_1 and data set_2 (2, 5). Strain names, sources, and available strain metadata are summarized in Data Set S1 in the supplemental material. Data set_1 comprised Illumina 101-bp paired-end genome sequence data from 95 ST131 strains isolated from 2000 to 2011, mostly in Europe and Oceania (2) (study accession no. ERP001354 and ERP004358). Data set_2 comprised Illumina 101-bp (76 samples), 76-bp (19 samples), and 50-bp (10 samples) paired-end whole-genome sequence data from 105 ST131 strains isolated from 1967 to 2011, mostly in North America (5) (study accession no. SRP027327). Additionally, reference strains of 11 published complete genomes were also used: namely, *E. coli* ST131 strains SE15, JJ1886, and EC958 plus non-ST131 B2 phylogenetic group *E. coli* strains CFT073, UTI89, E2348, ED1a, 536, S88, and APEC-01 and non-ST131 D phylogenetic group *E. coli* strain UMN026 (see Data Set S1). *E. coli* strain NA114 was excluded from the analysis due to poor assembly quality (2, 7, 26). To integrate reference genome data into our phylogenomic analyses, error-free 101-bp paired-end Illumina reads were simulated to 60× coverage with an insert size of 340 ± 40 bp as previously described (2).

### Quality control, *de novo* genome assembly, and variant detection.

Quality control (QC) was performed for all raw read data sets. Briefly, raw reads were analyzed using PRINSEQ v0.20.3 (27) and trimmed with a mean base pair quality score (*Q*) of ≥20 and a read length of ≥70% of the original read length. Additionally, it was necessary to correct 35 sets of raw read data from data set_2 that had heterogeneous Illumina encoding and/or erroneous paired-end length encoding (see Data Set S1 in the supplemental material). QC and assembly metrics for data set_1 have been previously reported by Petty et al. (2). Lastly, contaminant searches were performed for each sample using Kraken on a subset of 100,000 randomly chosen reads (28).

Quality-filtered Illumina paired-end reads were assembled *de novo* using Velvet v1.2.07 (29) with *k*-mer ranges of 45 to 85 for 101-bp reads, 29 to 61 for 76-bp reads, and 29 to 47 for 50-bp reads. An optimal *k*-mer value for each assembly was selected on the basis of best assembly metrics, including *N*_50_ (i.e., 50% of bases are incorporated in contigs of this length or above), number and size of contigs, number and continuity of uncalled bases, and peak coverage. Contigs that were ≥200 bp at an optimal *k*-mer were then ordered against *E. coli* EC958 (7) using Mauve v2.3.1 (30). QC and assembly statistics for data set_2 are summarized in the supplemental material.

Quality-filtered Illumina reads for data set_1 and data set_2, as well as error-free simulated reads of complete genomes, were mapped on the reference strain EC958 using SHRIMP v2.0 (31). Nesoni v0.108 (32) was used to call and annotate substitution-only SNPs, with a consensus cutoff and majority cutoff of 0.90 and 0.70, respectively. SNPs were also determined in parallel using the reference-free *k*-mer-based approach developed in kSNP v2.0 (33). Default parameters as well as a *k*-mer value of 19 selected as the optimal value predicted by the kSNP-associated Kchooser script were applied.

### Exclusion of suboptimal genome data sets.

We devised a statistical approach that excluded outliers based on several non-Gaussian metrics that could be determined from mapped and assembled Illumina genome data (see Data Set S1 in the supplemental material). Specifically we examined five metrics: (i) sequence coverage, (ii) number of unmapped bases (in the mapping reference EC958 genome), (iii) number of uncalled bases due to low coverage or mixed-base calls), (iv) the number of scaffolds that were ≥200 bp, and (v) estimated genome size. Metrics i, ii, and iii were based on read mapping data, and metrics iv and v were based on assemblies. Suboptimal genomes were discriminated quantitatively on the basis of metrics iii, iv, and v, and a total of 14 outliers were identified (based on upper and lower cutoffs at the quartile 3 + 1.5 interquartile range and the quartile 1 to 1.5 interquartile range cutoffs, respectively). Metric i did not identify any outliers with low sequence coverage, and outliers with high sequence coverage were not omitted, whereas metric ii did not discriminate any outliers. This additional QC process resulted in the exclusion of genome data from the following strains: CD301, CD436, JJ1901, JJ1996, JJ2007, JJ2041, JJ2050, JJ2243, JJ2441, JJ2555, MH17102, QU300, QUC02, and ZH193. The R scripts used are available on github at https://github.com/BeatsonLab-MicrobialGenomics/ST131_200_Rscripts. A final data set of 188 ST131 genomes, comprising 185 strains from data set_1 and data set_2 combined, as well as three complete genomes of EC958, JJ1886, and SE15) were chosen for further study after excluding the 14 genomes with suboptimal data quality.

### Recombination detection.

To avoid distortion of the phylogenetic signal caused by SNPs acquired through recombination, we used the Bayesian clustering algorithm BRATNextGen (34) to detect recombinant regions among the combined data set. Similar to our previous work (2), we used as an input an SNP-based multiple-genome alignment composed of each strain-specific pseudogenome built by integrating the SNPs predicted for each strain to the reference genome of EC958. To help with the identification of underlying clusters of strains, BRATNextGen initially computes a hierarchical clustering tree relative to the proportion of ancestral sequences shared between all strains. A segregation cutoff of 0.12 was then specified to separate each previously identified ST131 clade (clades A, B, and C) and non-ST131 strains into distinct clusters. Recombination was then evaluated within and between each cluster with the convergence approximated using 20 iterations of the learning algorithm. Significance was estimated using 100 permutations with a statistical significance threshold of 0.05. Using the same initial data set, recombination analysis was also carried out using Gubbins (35), an independent method of recombination detection.

### Phylogenetic analysis.

SNPs identified through reference-based mapping for the 188 ST131 strains were used to build phylogenies using maximum likelihood (ML), prior to and after removal of SNPs associated with recombinant regions. Phylogenetic trees were generated with RAxML v7.2.8 (36) using the general time-reversible (GTR) GAMMA model of among-site rate variation (ASRV), and validated using 1,000 bootstrap repetitions to assess nodal support. Additionally, reference-free *k*-mer-based phylogenetic trees were constructed using kSNP v2.0 with default parameters (33) and genome assemblies as an input. A *k*-mer value of 19 was selected as the optimal value predicted by the kSNP-associated Kchooser script. All trees were then viewed using Figtree v1.4.0 (37) or EvolView (38), and further compared using the Tanglegram algorithm of Dendroscope v3.2.10 (39), which generates two rectangular phylograms to allow comparison of bifurcating trees.

### Bayesian temporal and geographical analysis.

Preliminary estimation of the underlying temporal signal of our data was obtained by performing a regression analysis between the root-to-tip genetic distance extracted from the recombination-free maximum likelihood tree, the isolation year, and lineage information for each sequence, as implemented in Path-O-Gen v1.4 (22). To further investigate the divergence of clade C from clade B, we performed a temporal analysis on the 3,779-bp nonrecombinant SNPs of the 172 clade B and C strains using BEAST 2.0 (18), a Bayesian phylogenetic inference software, which can estimate the dating of emergence of distinct lineages. We compared multiple combinations of the molecular clock model (strict, constant relaxed log normal, and exponential relaxed log normal), substitution model (Hasegawa, Kishino, and Yano [HKY] model and GTR), and population size change model (coalescent constant, exponential growth, Bayesian skyline, and extended Bayesian skyline). Markov chain Monte Carlo (MCMC) generations for each analysis were conducted in triplicate for 100 million steps, sampling every 1,000 steps, to ensure convergence. Replicate analyses were then combined with LogCombiner, with a 10% burn-in. The GTR nucleotide substitution model was preferred over the HKY model, and was used with four discrete gamma-distributed rate categories and a default gamma prior distribution of 1. The uncorrelated log normal clock model consistently gave better support based on the Bayes factor and Akaike’s information criterion-based (AICM) analyses, compared to a strict clock model. The Bayesian skyline population tree model was chosen as the best-fitting tree model. Maximum clade credibility (MCC) trees reporting mean values with a posterior probability limit set at 0.5 were then created using TreeAnnotator.

In order to adequately investigate the biogeographical history of our ST131 collection, we evaluated potential bias in the geographical origin of strains, which could negatively impact our predictions. Statistical significance of the geographical origin distribution in clade B, C1, and C2 was assessed by chi-square test with Bonferroni correction for multiple comparisons. Overrepresented countries were randomly subsampled down to 15 representative sequences, while countries with fewer than 5 representatives had to be excluded from the analysis (South Korea and Portugal). Overall, we constructed 10 independent randomly subsampled data sets with 85 isolates representing 7 countries, each with 5 to 15 representative sequences. Reconstruction of possible ancestral geographical states was then performed using BEAST 1.8.2 on each subsampled data set. In addition to the previous parameters selected for the temporal analysis, a symmetric substitution model, a Bayesian stochastic search variable selection (BSSVS) model, and a strict clock for discrete locations were chosen for the phylogeographical analysis. MCMC generations were conducted for 100,000,000 steps, sampling every 10,000 steps. MCC trees were then generated using TreeAnnotator for each run with a posterior probability limit set at 0.5. Location posterior probabilities of the most recent common ancestor (MRCA) were then collated for clades B and C and for clade C only.

### Genomic comparisons and *in silico* genotyping.

Comparative genomic analyses were conducted using a combination of tools, namely, Artemis, Artemis Comparison Tool (40), and Mauve (30). Graphical representations showing the presence, absence, or variation of mobile genetic elements (MGEs) or other regions of interest, virulence factor genes, and antibiotic resistance genes were carried out using BLASTn and read-mapping information as implemented in the SeqFindR visualization tool (41). Regions of interest previously described in the genome of ST131 reference strain EC958 (2, 7) and virulence factors, including autotransporters, fimbriae, iron uptake, toxins, UPEC-specific genes, and other virulence genes, were screened in all ST131 strains with SeqFindR using a cutoff of ≥95% nucleotide identity over the whole length compared to the assembly or the consensus generated from mapping. Additionally, the prevalence of antibiotic resistance-associated genes was also investigated using Srst2 (42) against the ARGannot database, with a minimum depth of 15× read coverage.

## SUPPLEMENTAL MATERIAL

Data Set S1 Supplemental tables. Included is information on (i) strain collection and metadata, (ii) recombination regions based on the EC958 reference genome, (iii) EC958 and JJ1886 genomic island comparison, (iv) a summary of BEAST analysis results, (v) probabilities of all ancestral locations for clades B and C and C alone across 10 randomly subsampled subsets, (vi) the distribution of antibiotic resistance genes identified in ST131, (vii) a list of the complete genomes used in this study, and (viii) the quality control and assembly metrics for data set_2. Download Data Set S1, XLSX file, 0.3 MB

Figure S1  Quality assessment of genome data using *de novo* genome assembly and short-read mapping metrics. Box plots display three reference-based short-read mapping metrics used to identify genome data of suboptimal quality within data set_2, namely, sequence coverage relative to the reference EC958, number of unmapped bases (in the mapping reference), and number of uncalled bases (due to low coverage or mixed-base calls), as well as two *de novo* assembly metrics, namely, number of scaffolds that are ≥200 bp and estimated genome size (in base pairs). Outlier strains identified from the analysis are marked in orange. Download Figure S1, PDF file, 0.2 MB

Figure S2  Phylogenetic trees pre- and postrecombination filtering. Phylogenetic relationships of ST131 strains are shown using maximum likelihood (ML) phylograms rooted using the outgroup phylogroup D strain *E. coli* UMN026; branch lengths correspond to difference in the number of substitution-only SNPs (as shown by the scale shown at the bottom of each tree). Substitution-only SNPs were determined by read mapping using *E. coli* EC958 as the reference. The taxon labels for ST131 strains in this study are colored red (clade A), orange (clade B), pink (intermediate strains between clades B and C), and green (clade C). Red circles indicate support nodes of at least 95% bootstrap support from 1,000 replicates. (A) Maximum likelihood (ML) phylogram built from 21,373 substitution-only SNPs. (B) ML phylogram built from 5,471 substitution-only SNPs, excluding recombinant regions as defined by BRATNextGen analysis. Download Figure S2, PDF file, 1.2 MB

Figure S3  Phylogenetic tree based on alignment-free SNP matrix. The phylogenetic relationships of ST131 strains are shown using a midpoint-rooted maximum likelihood (ML) phylogram generated by RAxML with 1,000 bootstrap replicates from the core SNP matrix as determined by the alignment-free approach in kSNP v2. The taxon labels for ST131 strains in this study are colored red (clade A), orange (clade B), pink (intermediate strains between clades B and C), and green (clade C). Download Figure S3, PDF file, 0.02 MB

Figure S4  Recombination regions predicted in ST131 by BRATNextGen. Homologous recombination regions of the 188 ST131 strains were predicted using BRATNextGen. Shown are the results from recombination inference of a multiple-pseudogenome alignment, which was created by integrating each strain-specific substitution SNP onto the reference EC958 core genome. A maximum likelihood (ML) phylogram of the 188 ST131 strains (based on 5,471 recombination-free substitution SNPs) is shown on the left. The scale bar indicates the number of substitution SNPs. Inferred recombination segments for each strain are depicted as solid blocks colored according to their clade designation as follows: namely, clade A in red, clade B in orange, intermediate strains between clades B and C in pink, and clade C in green. The *x* axis at the bottom of the matrix indicates the genome position (in megabases) relative to the EC958 reference genome. The top row indicates regions of interest (ROI) in blue, which include MGEs, regions of difference (ROD), O-antigen clusters, and others. Download Figure S4, PDF file, 0.5 MB

Figure S5  Recombination regions predicted in ST131 by Gubbins. Regions of interest previously described in the genome of ST131 reference strain EC958 are shown along the *x* axis with strain identifiers on the *y* axis in the same order as the phylogenetic tree shown in Fig. S2B in the supplemental material. On the left panel, a phylogenetic tree based on nonrecombinant regions is colored as follows: namely, clade A in red, clade B in orange, clades B0 and C0 in pink, and clade C in green. The right panel shows the recombination profile of each strain horizontally. Recombinant regions found in at least two strains are shown in red and unique recombinant regions in blue. Download Figure S5, PDF file, 0.6 MB

Figure S6  Regions of interest in the accessory genomes of ST131 strains. Screening for the presence/absence of regions of interest was performed using the BLAST-based visualization tool SeqFindR (41). Regions of interest previously described in the genome of ST131 reference strain EC958 are shown along the *x* axis with strain identifiers on the *y* axis in the same order as the phylogenetic tree shown in Fig. S2B in the supplemental material. All regions are plotted in the same order as they are found along the EC958 reference genome, with their name labeled on top of the matrix. Black shading indicates a match of ≥95% nucleotide identity when comparing the query sequence to the assemblies or the consensus generated from read mapping. Download Figure S6, PDF file, 1.2 MB

Figure S7  Prevalence of representative virulence factors in ST131 strains. Screening for the presence/absence of virulence factors was performed using the BLAST-based visualization tool SeqFindR (41). Virulence genes are shown along the *x* axis with strain identifiers on the *y* axis in the same order as the phylogenetic tree shown on Fig. S2B in the supplemental material. Virulence genes are listed in groups and correspond to (i) autotransporters, (ii) chaperone usher fimbriae, (iii) iron uptake, (iv) other virulence genes, (v) toxins, or (vi) UPEC-specific genes. Black shading shows a match of ≥95% nucleotide identity when comparing the query sequence to the assemblies or the consensus generated from read mapping. Download Figure S7, PDF file, 2.4 MB

Figure S8  Temporal signal for clade B and C strains. The correlation between the root-to-tip distances and the isolation time was estimated using Path-O-Gen for clade B and C strains (a) and clade C strains only (b). Download Figure S8, PDF file, 0.3 MB

Figure S9  Maximum clade credibility tree of ST131 multidrug-resistant clades B and C with error bars. A time-calibrated phylogeny was reconstructed using BEAST 2.0 based on 3,779-bp nonrecombinant SNPs for the 172 clade B and C strains with the GTR substitution model, a constant relaxed clock model, and the Bayesian skyline population tree model. The maximum clade credibility tree is colored according to clade origin as shown on the right, with B in orange, intermediate B0 and C0 in pink, C1 in light green, and C2 in dark green. The *x* axis indicates emergence time estimates of the corresponding strains. Node bars represent 95% highest posterior density intervals. Download Figure S9, PDF file, 1.6 MB

## References

[1] Nicolas-Chanoine MH, Blanco J, Leflon-Guibout V, Demarty R, Alonso MP, Caniça MM, Park YJ, Lavigne JP, Pitout J, Johnson JR 2008 Intercontinental emergence of *Escherichia coli* clone O25:H4-ST131 producing CTX-M-15. J Antimicrob Chemother 61:273–281. doi:10.1093/jac/dkm464. 18077311

[2] Petty NK, Ben Zakour NL, Stanton-Cook M, Skippington E, Totsika M, Forde BM, Phan MD, Gomes Moriel D, Peters KM, Davies M, Rogers BA, Dougan G, Rodriguez-Baño J, Pascual A, Pitout JD, Upton M, Paterson DL, Walsh TR, Schembri MA, Beatson SA 2014 Global dissemination of a multidrug resistant Escherichia coli clone. Proc Natl Acad Sci U S A 111:5694–5699. doi:10.1073/pnas.1322678111. 24706808 PMC3992628

[3] Totsika M, Beatson SA, Sarkar S, Phan MD, Petty NK, Bachmann N, Szubert M, Sidjabat HE, Paterson DL, Upton M, Schembri MA 2011 Insights into a multidrug resistant Escherichia coli pathogen of the globally disseminated ST131 lineage: genome analysis and virulence mechanisms. PLoS One 6:e00347-16. doi:10.1371/journal.pone.0026578. PMC3203889 22053197

[4] Totsika M, Moriel DG, Idris A, Rogers BA, Wurpel DJ, Phan MD, Paterson DL, Schembri MA 2012 Uropathogenic Escherichia coli mediated urinary tract infection. Curr Drug Targets 13:1386–1399. doi:10.2174/138945012803530206. 22664092

[5] Price LB, Johnson JR, Aziz M, Clabots C, Johnston B, Tchesnokova V, Nordstrom L, Billig M, Chattopadhyay S, Stegger M, Andersen PS, Pearson T, Riddell K, Rogers P, Scholes D, Kahl B, Keim P, Sokurenko EV 2013 The epidemic of extended-spectrum-beta-lactamase-producing Escherichia coli ST131 is driven by a single highly pathogenic subclone, H30-Rx. mBio 4:e00377-13. doi:10.1128/mBio.00377-13. 24345742 PMC3870262

[6] Johnson JR, Tchesnokova V, Johnston B, Clabots C, Roberts PL, Billig M, Riddell K, Rogers P, Qin X, Butler-Wu S, Price LB, Aziz M, Nicolas-Chanoine MH, Debroy C, Robicsek A, Hansen G, Urban C, Platell J, Trott DJ, Zhanel G, Weissman SJ, Cookson BT, Fang FC, Limaye AP, Scholes D, Chattopadhyay S, Hooper DC, Sokurenko EV 2013 Abrupt emergence of a single dominant multidrug-resistant strain of Escherichia coli. J Infect Dis 207:919–928. doi:10.1093/infdis/jis933. 23288927 PMC3571447

[7] Forde BM, Ben Zakour NL, Stanton-Cook M, Phan MD, Totsika M, Peters KM, Chan KG, Schembri MA, Upton M, Beatson SA 2014 The complete genome sequence of Escherichia coli EC958: a high quality reference sequence for the globally disseminated multidrug resistant E. coli O25b:H4-ST131 clone. PLoS One 9:e00347-16. doi:10.1371/journal.pone.0104400. PMC4134206 25126841

[8] Phan MD, Peters KM, Sarkar S, Lukowski SW, Allsopp LP, Gomes Moriel D, Achard ME, Totsika M, Marshall VM, Upton M, Beatson SA, Schembri MA 2013 The serum resistome of a globally disseminated multidrug resistant uropathogenic Escherichia coli clone. PLoS Genet 9:e00347-16. doi:10.1371/journal.pgen.1003834. PMC3789825 24098145

[9] Forde BM, Phan MD, Gawthorne JA, Ashcroft MM, Stanton-Cook M, Sarkar S, Peters KM, Chan KG, Chong TM, Yin WF, Upton M, Schembri MA, Beatson SA 2015 Lineage-specific methyltransferases define the methylome of the globally disseminated Escherichia coli ST131 clone. mBio 6:e01602-15. doi:10.1128/mBio.01602-15. 26578678 PMC4659465

[10] Kakkanat A, Totsika M, Schaale K, Duell BL, Lo AW, Phan MD, Moriel DG, Beatson SA, Sweet MJ, Ulett GC, Schembri MA 2015 The role of H4 flagella in Escherichia coli ST131 virulence. Sci Rep 5:16149. doi:10.1038/srep16149. 26548325 PMC4637896

[11] Phan MD, Forde BM, Peters KM, Sarkar S, Hancock S, Stanton-Cook M, Ben Zakour NL, Upton M, Beatson SA, Schembri MA 2015 Molecular characterization of a multidrug resistance IncF plasmid from the globally disseminated Escherichia coli ST131 clone. PLoS One 10:e00347-16. doi:10.1371/journal.pone.0122369. PMC4398462 25875675

[12] Phan MD, Peters KM, Sarkar S, Forde BM, Lo AW, Stanton-Cook M, Roberts LW, Upton M, Beatson SA, Schembri MA 2015 Third-generation cephalosporin resistance conferred by a chromosomally encoded blaCMY-23 gene in the Escherichia coli ST131 reference strain EC958. J Antimicrob Chemother 70:1969–1972. doi:10.1093/jac/dkv066. 25786480

[13] Coelho A, Mora A, Mamani R, López C, González-López JJ, Larrosa MN, Quintero-Zarate JN, Dahbi G, Herrera A, Blanco JE, Blanco M, Alonso MP, Prats G, Blanco J 2011 Spread of Escherichia coli O25b:H4-B2-ST131 producing CTX-M-15 and SHV-12 with high virulence gene content in Barcelona (Spain). J Antimicrob Chemother 66:517–526. doi:10.1093/jac/dkq491. 21177675

[14] Johnson JR, Johnston B, Clabots C, Kuskowski MA, Castanheira M 2010 Escherichia coli sequence type ST131 as the major cause of serious multidrug-resistant E. coli infections in the United States. Clin Infect Dis 51:286–294. doi:10.1086/653932. 20572763

[15] Johnson JR, Menard M, Johnston B, Kuskowski MA, Nichol K, Zhanel GG 2009 Epidemic clonal groups of Escherichia coli as a cause of antimicrobial-resistant urinary tract infections in Canada, 2002 to 2004. Antimicrob Agents Chemother 53:2733–2739. doi:10.1128/AAC.00297-09. 19398649 PMC2704706

[16] Lanza VF, de Toro M, Garcillán-Barcia MP, Mora A, Blanco J, Coque TM, de la Cruz F 2014 Plasmid flux in Escherichia coli ST131 sublineages, analyzed by plasmid constellation network (PLACNET), a new method for plasmid reconstruction from whole genome sequences. PLoS Genet 10:e00347-16. doi:10.1371/journal.pgen.1004766. PMC4270462 25522143

[17] Stoesser N, Sheppard AE, Pankhurst L, De Maio N, Moore CE, Sebra R, Turner P, Anson LW, Kasarskis A, Batty EM, Kos V, Wilson DJ, Phetsouvanh R, Wyllie D, Sokurenko E, Manges AR, Johnson TJ, Price LB, Peto TEA, Johnson JR, Didelot X, Walker AS, Crook DW, Modernizing Medical Microbiology Informatics Group (MMMIG) 2016 Evolutionary history of the global emergence of the *Escherichia coli* epidemic clone ST131. mBio 7(2):e02162-15. doi:10.1128/mBio.02162-15. PMC4807372 27006459

[18] Bouckaert R, Heled J, Kühnert D, Vaughan T, Wu CH, Xie D, Suchard MA, Rambaut A, Drummond AJ 2014 BEAST 2: a software platform for Bayesian evolutionary analysis. PLoS Comput Biol 10:e00347-16. doi:10.1371/journal.pcbi.1003537. PMC3985171 24722319

[19] Da Cunha V, Davies MR, Douarre PE, Rosinski-Chupin I, Margarit I, Spinali S, Perkins T, Lechat P, Dmytruk N, Sauvage E, Ma L, Romi B, Tichit M, Lopez-Sanchez MJ, Descorps-Declere S, Souche E, Buchrieser C, Trieu-Cuot P, Moszer I, Clermont D, Maione D, Bouchier C, McMillan DJ, Parkhill J, Telford JL, Dougan G, Walker MJ, DEVANI Consortium, Holden MT, Poyart C, Glaser P 2014 Streptococcus agalactiae clones infecting humans were selected and fixed through the extensive use of tetracycline. Nat Commun 5:4544. doi:10.1038/ncomms5544. 25088811 PMC4538795

[20] Holden MT, Hsu LY, Kurt K, Weinert LA, Mather AE, Harris SR, Strommenger B, Layer F, Witte W, de Lencastre H, Skov R, Westh H, Zemlicková H, Coombs G, Kearns AM, Hill RL, Edgeworth J, Gould I, Gant V, Cooke J, Edwards GF, McAdam PR, Templeton KE, McCann A, Zhou Z, Castillo-Ramirez S, Feil EJ, Hudson LO, Enright MC, Balloux F, Aanensen DM, Spratt BG, Fitzgerald JR, Parkhill J, Achtman M, Bentley SD, Nubel U 2013 A genomic portrait of the emergence, evolution, and global spread of a methicillin-resistant Staphylococcus aureus pandemic. Genome Res 23:653–664. doi:10.1101/gr.147710.112. 23299977 PMC3613582

[21] Andersen PS, Stegger M, Aziz M, Contente-Cuomo T, Gibbons HS, Keim P, Sokurenko EV, Johnson JR, Price LB 2013 Complete genome sequence of the epidemic and highly virulent CTX-M-15-producing H30-Rx subclone of Escherichia coli ST131. Genome Announc 1:e00988-13. doi:10.1128/genomeA.00988-13. 24309736 PMC3853059

[22] Rambaut A 2013 Path-O-Gen. http://tree.bio.ed.ac.uk/software/pathogen/. Accessed 8 April 2015.

[23] Holt KE, Thieu Nga TV, Thanh DP, Vinh H, Kim DW, Vu Tra MP, Campbell JI, Hoang NV, Vinh NT, Minh PV, Thuy CT, Nga TT, Thompson C, Dung TT, Nhu NT, Vinh PV, Tuyet PT, Phuc HL, Lien NT, Phu BD, Ai NT, Tien NM, Dong N, Parry CM, Hien TT, Farrar JJ, Parkhill J, Dougan G, Thomson NR, Baker S 2013 Tracking the establishment of local endemic populations of an emergent enteric pathogen. Proc Natl Acad Sci U S A 110:17522–17527. doi:10.1073/pnas.1308632110. 24082120 PMC3808646

[24] Kaitin KI, Richard BW, Lasagna L 1987 Trends in drug development: the 1985–86 new drug approvals. J Clin Pharmacol 27:542–548. doi:10.1002/j.1552-4604.1987.tb03064.x. 3655005

[25] Paul S, Linardopoulou EV, Billig M, Tchesnokova V, Price LB, Johnson JR, Chattopadhyay S, Sokurenko EV 2013 Role of homologous recombination in adaptive diversification of extraintestinal Escherichia coli. J Bacteriol 195:231–242. doi:10.1128/JB.01524-12. 23123908 PMC3553836

[26] Avasthi TS, Kumar N, Baddam R, Hussain A, Nandanwar N, Jadhav S, Ahmed N 2011 Genome of multidrug-resistant uropathogenic Escherichia coli strain NA114 from India. J Bacteriol 193:4272–4273. doi:10.1128/JB.05413-11. 21685291 PMC3147708

[27] Schmieder R, Edwards R 2011 Quality control and preprocessing of metagenomic datasets. Bioinformatics 27:863–864. doi:10.1093/bioinformatics/btr026. 21278185 PMC3051327

[28] Davis MP, van Dongen S, Abreu-Goodger C, Bartonicek N, Enright AJ 2013 Kraken: a set of tools for quality control and analysis of high-throughput sequence data. Methods 63:41–49. doi:10.1016/j.ymeth.2013.06.027. 23816787 PMC3991327

[29] Zerbino DR, Birney E 2008 Velvet: algorithms for de novo short read assembly using de Bruijn graphs. Genome Res 18:821–829. doi:10.1101/gr.074492.107. 18349386 PMC2336801

[30] Darling AE, Mau B, Perna NT 2010 progressiveMauve: multiple genome alignment with gene gain, loss and rearrangement. PLoS One 5:e00347-16. doi:10.1371/journal.pone.0011147. PMC2892488 20593022

[31] David M, Dzamba M, Lister D, Ilie L, Brudno M 2011 SHRiMP2: sensitive yet practical SHort Read mapping. BioInformatics 27:1011–1012. doi:10.1093/bioinformatics/btr046. 21278192

[32] Victorian-Bioinformatics-Consortium 2013 Nesoni downloads. http://www.vicbioinformatics.com/software.nesoni.shtml. Accessed 1 February 2015.

[33] Gardner SN, Hall BG 2013 When whole-genome alignments just won’t work: kSNP v2 software for alignment-free SNP discovery and phylogenetics of hundreds of microbial genomes. PLoS One 8:e00347-16. doi:10.1371/journal.pone.0081760. PMC3857212 24349125

[34] Marttinen P, Hanage WP, Croucher NJ, Connor TR, Harris SR, Bentley SD, Corander J 2012 Detection of recombination events in bacterial genomes from large population samples. Nucleic Acids Res 40:e6 doi:10.1093/nar/gkr928. 22064866 PMC3245952

[35] Croucher NJ, Page AJ, Connor TR, Delaney AJ, Keane JA, Bentley SD, Parkhill J, Harris SR 2015 Rapid phylogenetic analysis of large samples of recombinant bacterial whole genome sequences using Gubbins. Nucleic Acids Res 43:e15. doi:10.1093/nar/gku1196. 25414349 PMC4330336

[36] Stamatakis A 2006 RAxML-VI-HPC: maximum likelihood-based phylogenetic analyses with thousands of taxa and mixed models. Bioinformatics 22:2688–2690. doi:10.1093/bioinformatics/btl446. 16928733

[37] Rambaut A 2009 FigTree, a graphical viewer of phylogenetic trees. http://tree.bio.ed.ac.uk/software/figtree/. Accessed 1 February 2015.

[38] Zhang H, Gao S, Lercher MJ, Hu S, Chen WH 2012 EvolView, an online tool for visualizing, annotating and managing phylogenetic trees. Nucleic Acids Res 40:W569–W572. doi:10.1093/nar/gks576. 22695796 PMC3394307

[39] Huson DH, Scornavacca C 2012 Dendroscope 3: an interactive tool for rooted phylogenetic trees and networks.Syst Biol 61:1061–1067. doi:10.1093/sysbio/sys062. 22780991

[40] Carver T, Berriman M, Tivey A, Patel C, Böhme U, Barrell BG, Parkhill J, Rajandream MA 2008 Artemis and ACT: viewing, annotating and comparing sequences stored in a relational database. Bioinformatics 24:2672–2676. doi:10.1093/bioinformatics/btn529. 18845581 PMC2606163

[41] Stanton-Cook M, Ben Zakour NL, Alikhan NF, Beatson SA 2013 SeqFindR. http://mscook.github.io/SeqFindR/.

[42] Inouye M, Dashnow H, Raven LA, Schultz MB, Pope BJ, Tomita T, Zobel J, Holt KE 2014 SRST2: rapid genomic surveillance for public health and hospital microbiology labs. Genome Med 6:90. doi:10.1186/s13073-014-0090-6. 25422674 PMC4237778

