# Erratum for Ben Zakour et al., Sequential Acquisition of Virulence and Fluoroquinolone Resistance Has Shaped the Evolution of *Escherichia coli* ST131

**DOI:** 10.1128/mBio.00958-16

**Published:** 2016-06-28

**Authors:** Nouri L. Ben Zakour, Areej S. Alsheikh-Hussain, Melinda M. Ashcroft, Nguyen Thi Khanh Nhu, Leah W. Roberts, Mitchell Stanton-Cook, Mark A. Schembri, Scott A. Beatson

**Affiliations:** a Australian Infectious Diseases Research Centre, School of Chemistry and Molecular Biosciences, The University of Queensland, Brisbane, Australia; b Australian Centre for Ecogenomics, School of Chemistry and Molecular Biosciences, The University of Queensland, Brisbane, Australia

## ERRATUM

The article cited as reference 17 (N. Stoesser et al.) was originally available as a preprint in BioRxiv (http://dx.doi.org/10.1101/030668) on 6 November 2015. It was subsequently published in mBio while our article was in proof.

