# Supplementary figures and images for: Sequential Acquisition of Virulence and Fluoroquinolone Resistance Has Shaped the Evolution of Escherichia coli ST131
Source: mBio. 2016 Apr 26;7(2):e00347-16. doi: 10.1128/mBio.00347-16 (PMC4850260; doi:10.1128/mBio.00347-16)

Coverage

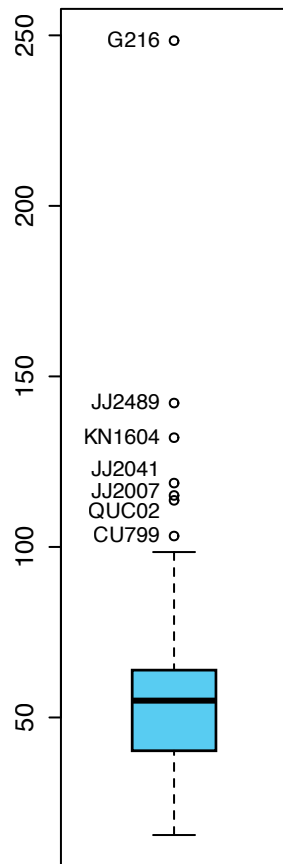

No. of unmapped bases

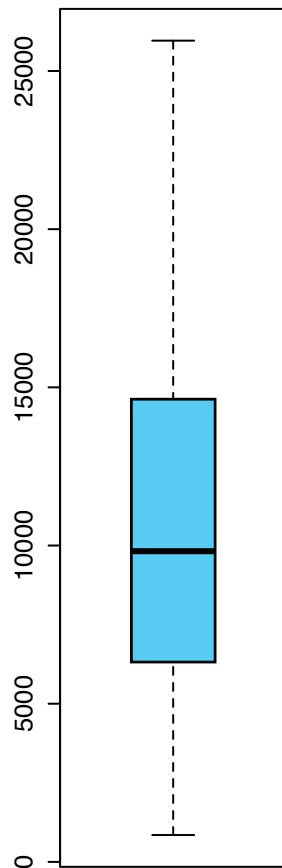

No. of uncalled bases

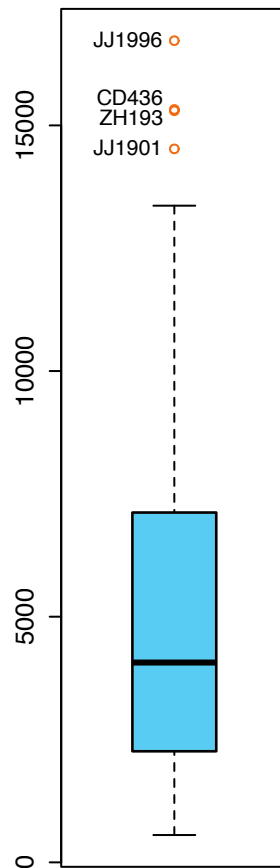

No. of scaffolds

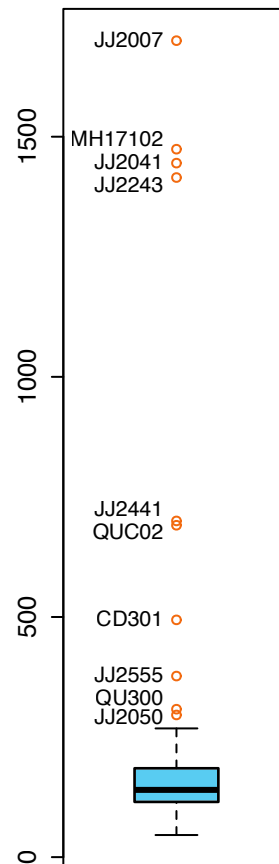

Genome size

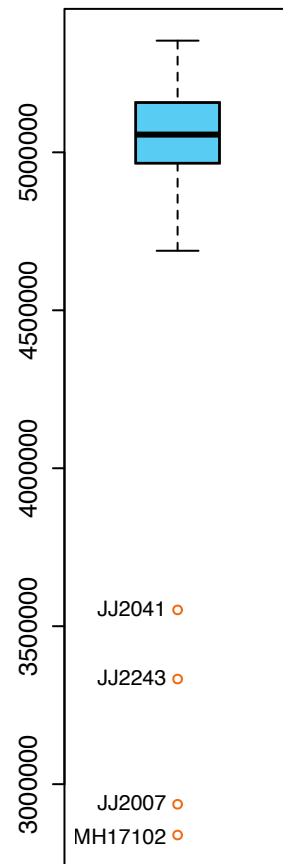

Supplement: Figure S1 — Quality assessment of genome data using de novo genome assembly and short-read mapping metrics. Box plots display three reference-based short-read mapping metrics used to identify genome data of suboptimal quality within data set_2, namely, sequence coverage relative to the reference EC958, number of unmapped bases (in the mapping reference), and number of uncalled bases (due to low coverage or mixed-base calls), as well as two de novo assembly metrics, namely, number of scaffolds that are ≥200 bp and estimated genome size (in base pairs). Outlier strains identified from the analysis are marked in orange. Download [file mbo002162781sf1.pdf]

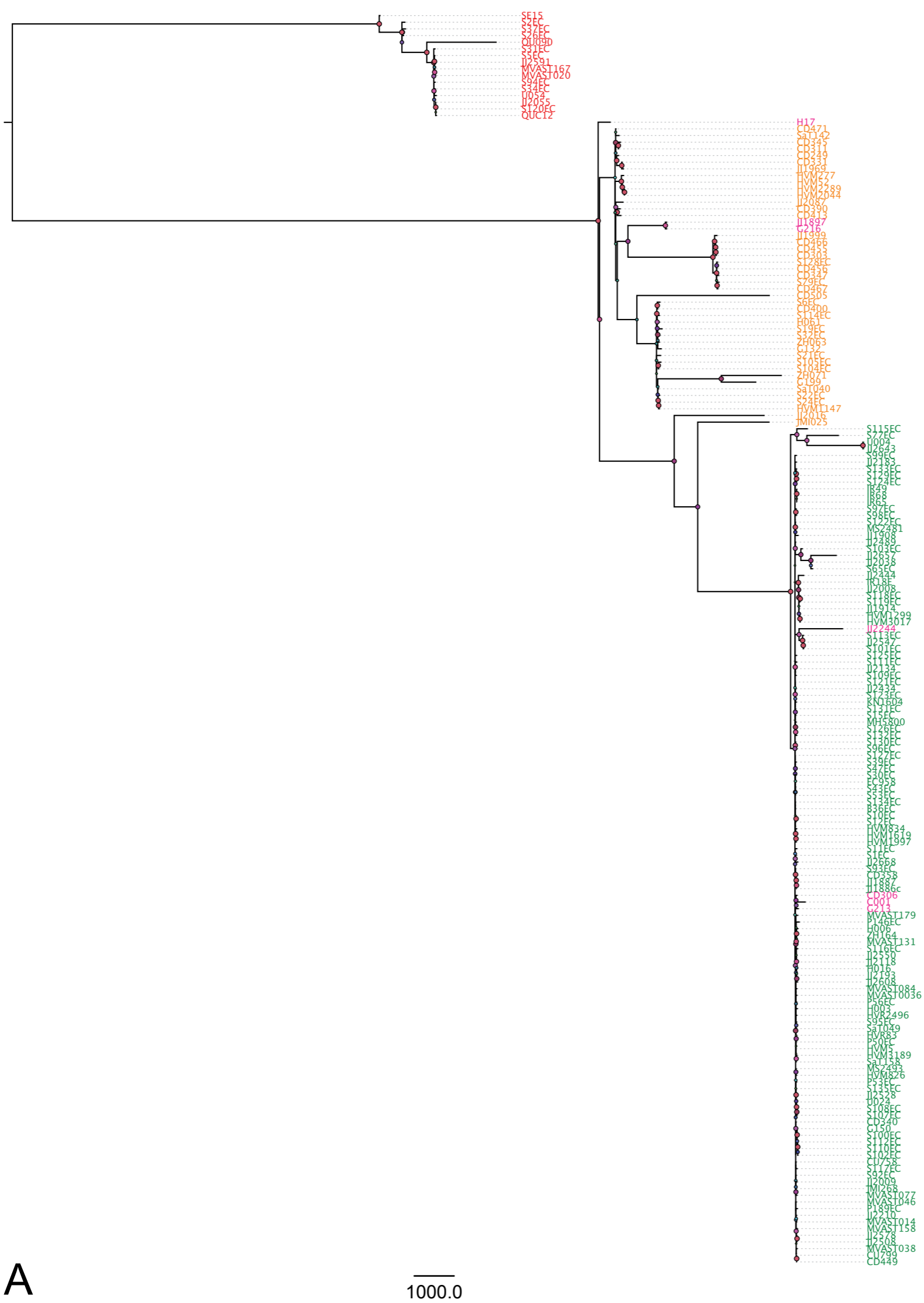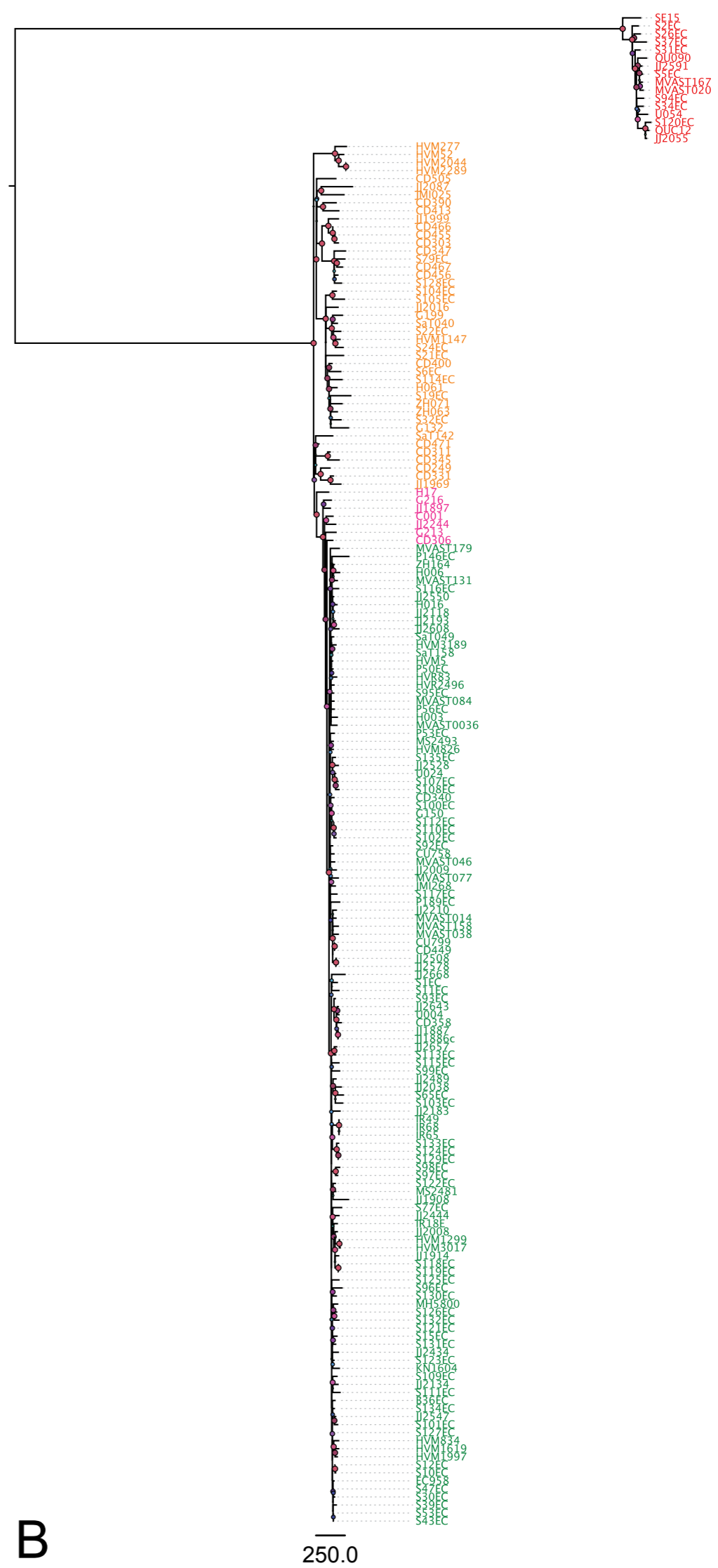

Supplement: Figure S2 — Phylogenetic trees pre- and postrecombination filtering. Phylogenetic relationships of ST131 strains are shown using maximum likelihood (ML) phylograms rooted using the outgroup phylogroup D strain E. coli UMN026; branch lengths correspond to difference in the number of substitution-only SNPs (as shown by the scale shown at the bottom of each tree). Substitution-only SNPs were determined by read mapping using E. coli EC958 as the reference. The taxon labels for ST131 strains in this study are colored red (clade A), orange (clade B), pink (intermediate strains between clades B and C), and green (clade C). Red circles indicate support nodes of at least 95% bootstrap support from 1,000 replicates. (A) Maximum likelihood (ML) phylogram built from 21,373 substitution-only SNPs. (B) ML phylogram built from 5,471 substitution-only SNPs, excluding recombinant regions as defined by BRATNextGen analysis. Download [file mbo002162781sf2.pdf]

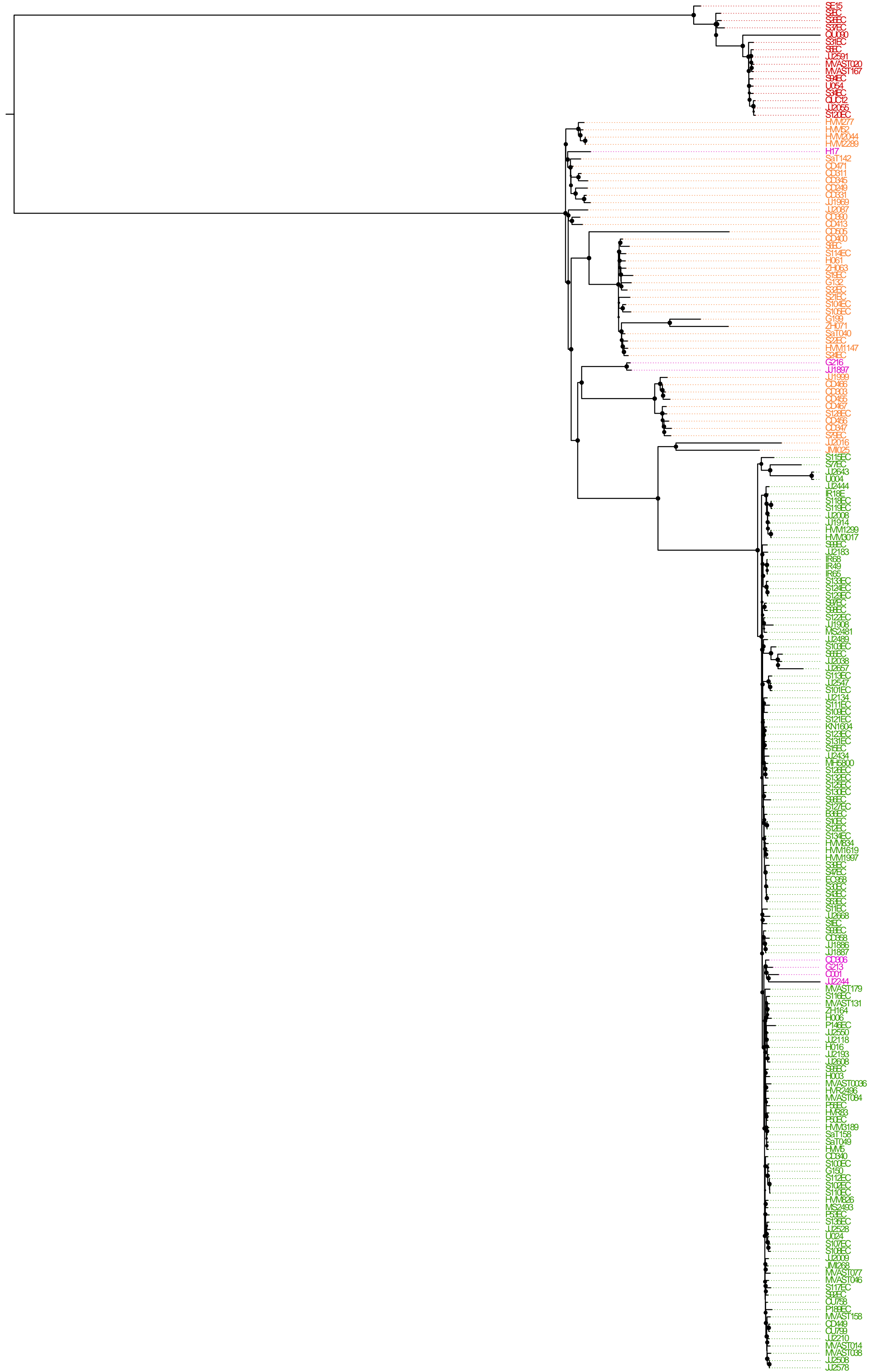

Supplement: Figure S3 — Phylogenetic tree based on alignment-free SNP matrix. The phylogenetic relationships of ST131 strains are shown using a midpoint-rooted maximum likelihood (ML) phylogram generated by RAxML with 1,000 bootstrap replicates from the core SNP matrix as determined by the alignment-free approach in kSNP v2. The taxon labels for ST131 strains in this study are colored red (clade A), orange (clade B), pink (intermediate strains between clades B and C), and green (clade C). Download [file mbo002162781sf3.pdf]

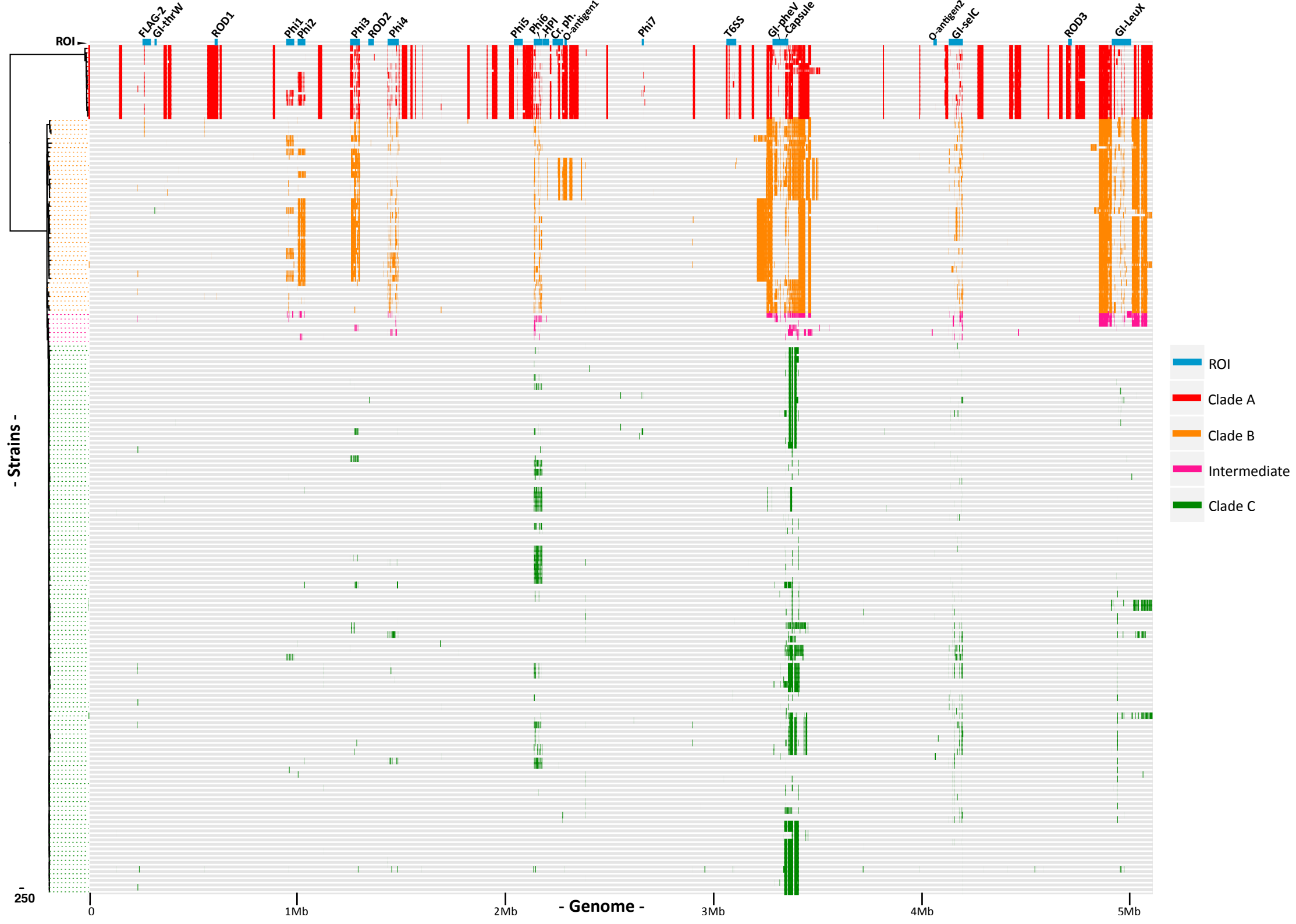

Supplement: Figure S4 — Recombination regions predicted in ST131 by BRATNextGen. Homologous recombination regions of the 188 ST131 strains were predicted using BRATNextGen. Shown are the results from recombination inference of a multiple-pseudogenome alignment, which was created by integrating each strain-specific substitution SNP onto the reference EC958 core genome. A maximum likelihood (ML) phylogram of the 188 ST131 strains (based on 5,471 recombination-free substitution SNPs) is shown on the left. The scale bar indicates the number of substitution SNPs. Inferred recombination segments for each strain are depicted as solid blocks colored according to their clade designation as follows: namely, clade A in red, clade B in orange, intermediate strains between clades B and C in pink, and clade C in green. The x axis at the bottom of the matrix indicates the genome position (in megabases) relative to the EC958 reference genome. The top row indicates regions of interest (ROI) in blue, which include MGEs, regions of difference (ROD), O-antigen clusters, and others. Download [file mbo002162781sf4.pdf]

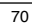

Supplement: Figure S5 — Recombination regions predicted in ST131 by Gubbins. Regions of interest previously described in the genome of ST131 reference strain EC958 are shown along the x axis with strain identifiers on the y axis in the same order as the phylogenetic tree shown in Fig. S2B in the supplemental material. On the left panel, a phylogenetic tree based on nonrecombinant regions is colored as follows: namely, clade A in red, clade B in orange, clades B0 and C0 in pink, and clade C in green. The right panel shows the recombination profile of each strain horizontally. Recombinant regions found in at least two strains are shown in red and unique recombinant regions in blue. Download [file mbo002162781sf5.pdf]

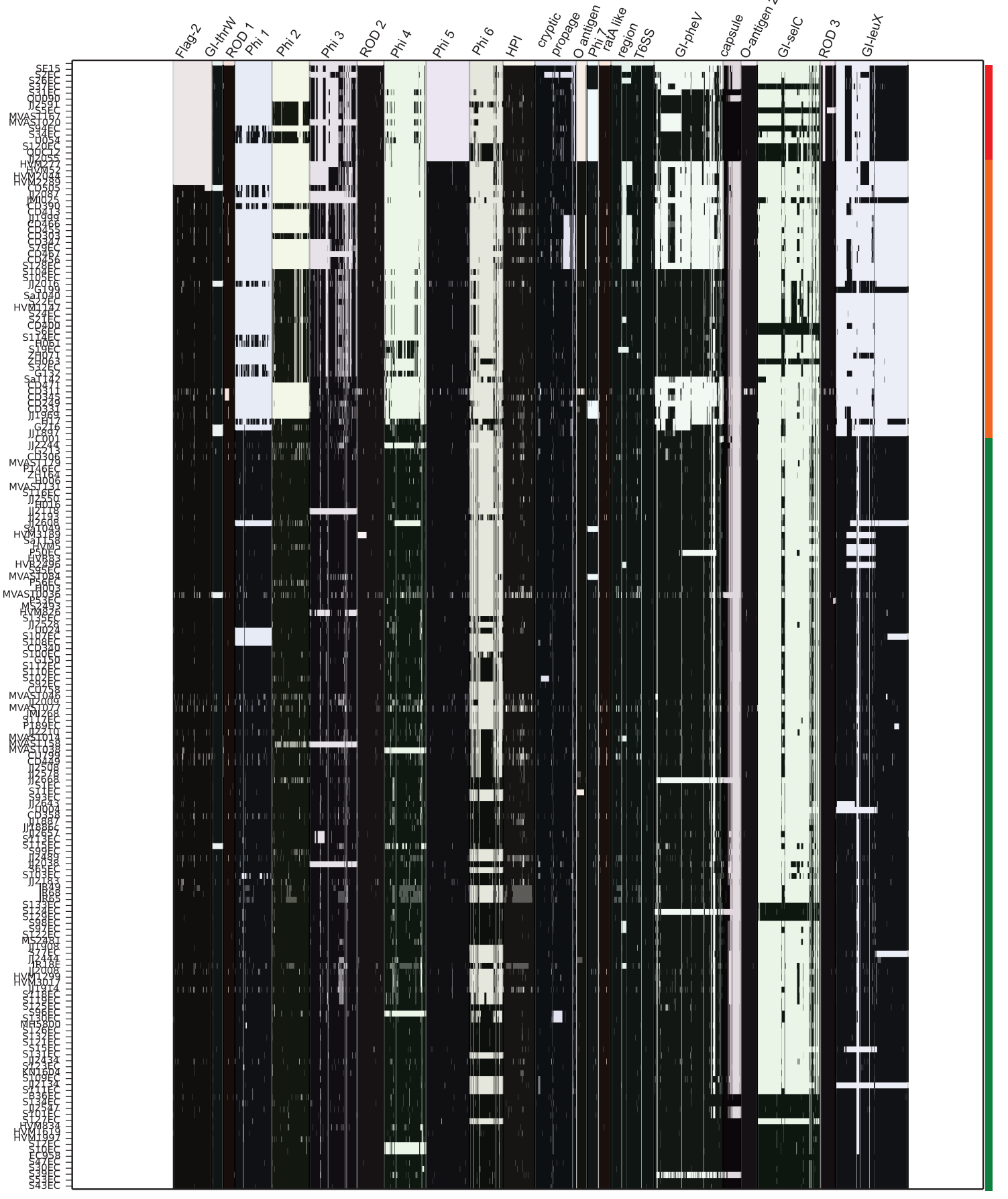

Supplement: Figure S6 — Regions of interest in the accessory genomes of ST131 strains. Screening for the presence/absence of regions of interest was performed using the BLAST-based visualization tool SeqFindR (41). Regions of interest previously described in the genome of ST131 reference strain EC958 are shown along the x axis with strain identifiers on the y axis in the same order as the phylogenetic tree shown in Fig. S2B in the supplemental material. All regions are plotted in the same order as they are found along the EC958 reference genome, with their name labeled on top of the matrix. Black shading indicates a match of ≥95% nucleotide identity when comparing the query sequence to the assemblies or the consensus generated from read mapping. Download [file mbo002162781sf6.pdf]

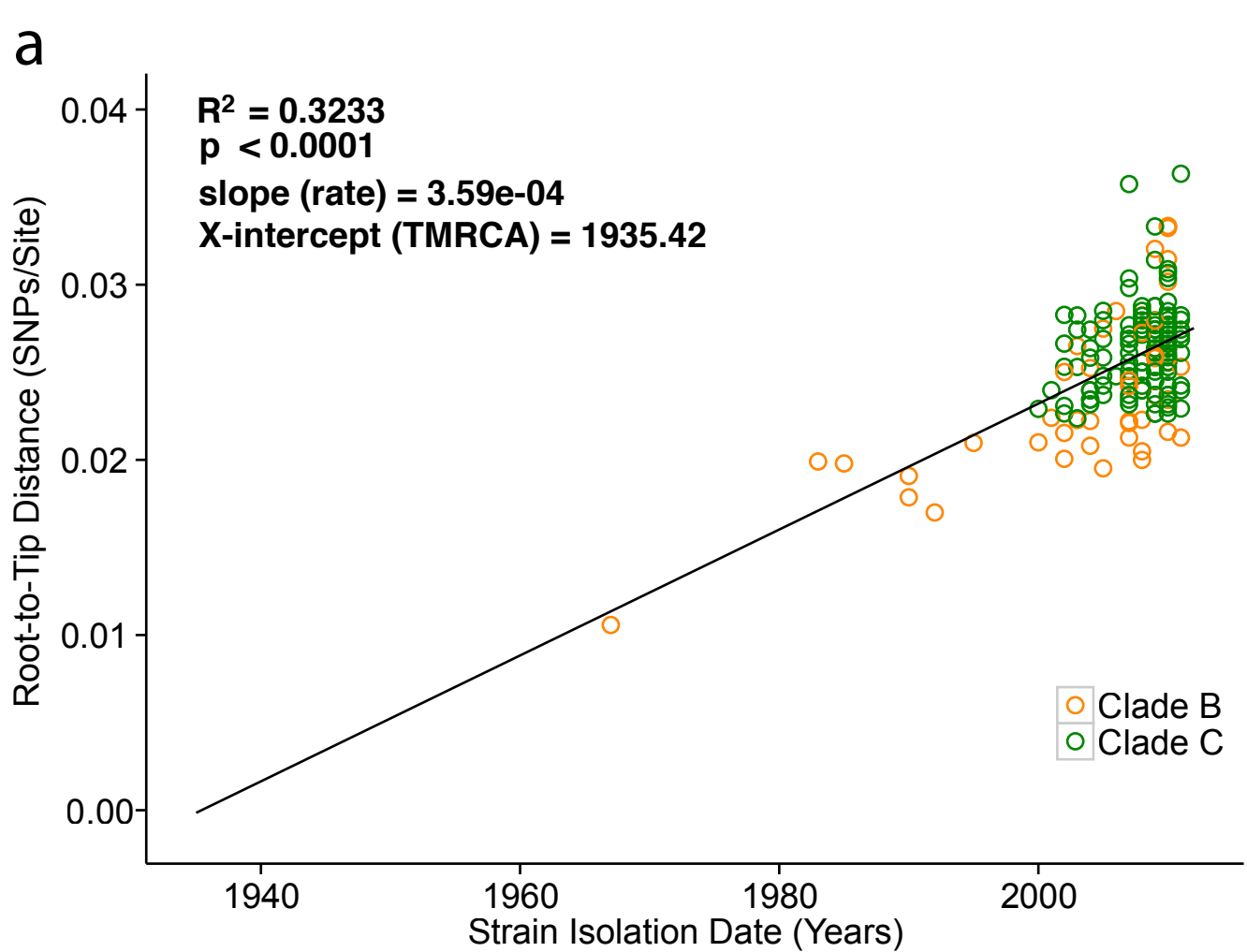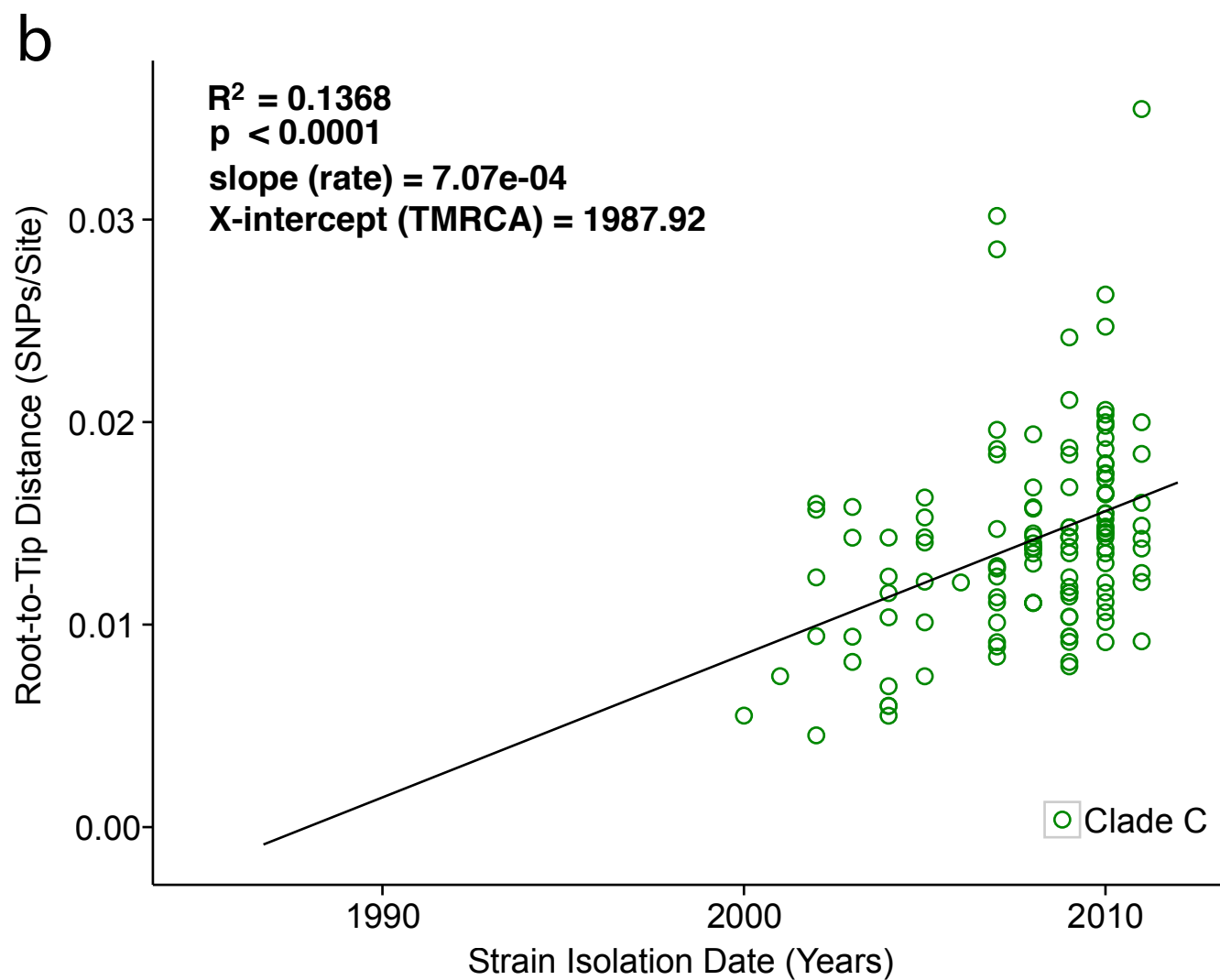

Supplement: Figure S8 — Temporal signal for clade B and C strains. The correlation between the root-to-tip distances and the isolation time was estimated using Path-O-Gen for clade B and C strains (a) and clade C strains only (b). Download [file mbo002162781sf8.pdf]

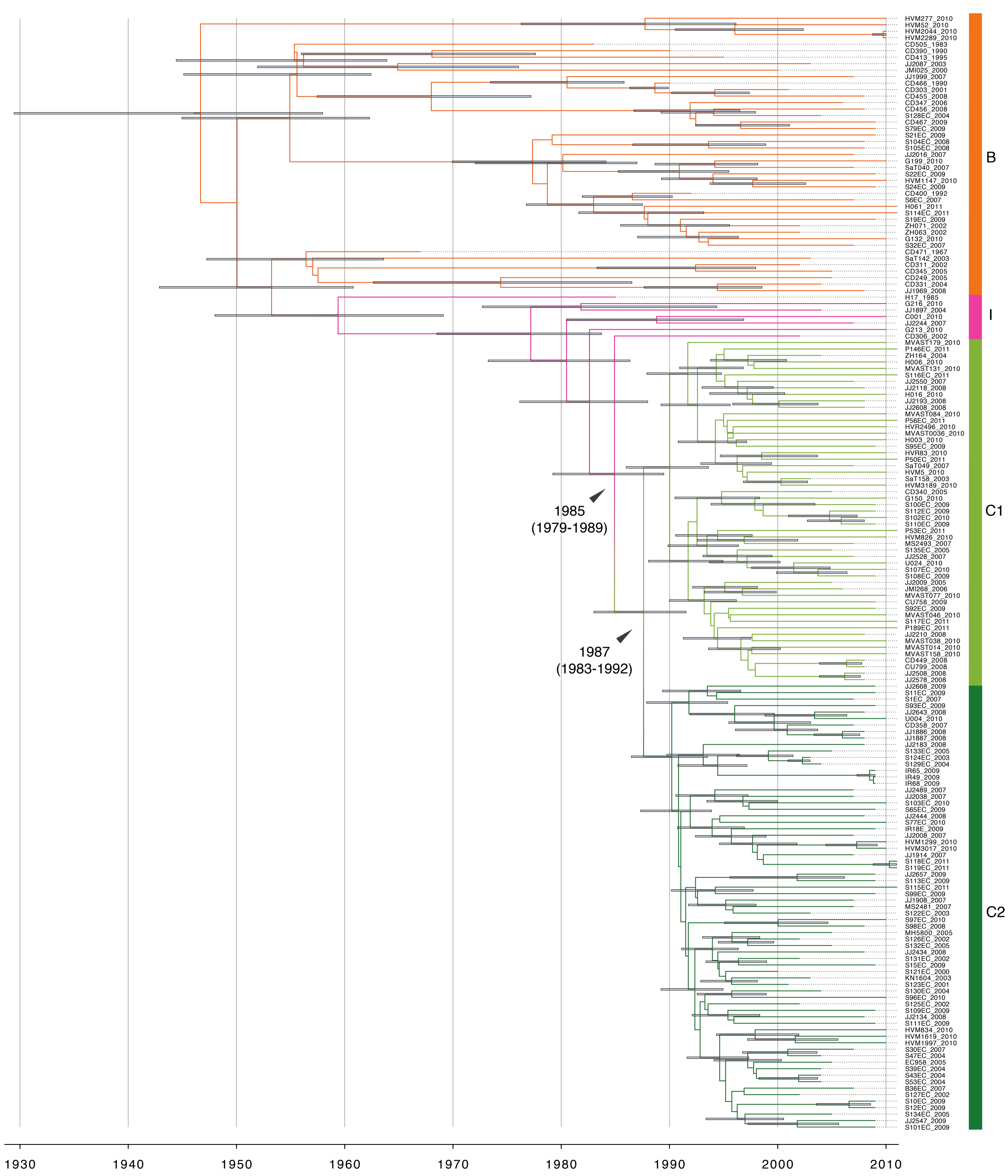

Supplement: Figure S9 — Maximum clade credibility tree of ST131 multidrug-resistant clades B and C with error bars. A time-calibrated phylogeny was reconstructed using BEAST 2.0 based on 3,779-bp nonrecombinant SNPs for the 172 clade B and C strains with the GTR substitution model, a constant relaxed clock model, and the Bayesian skyline population tree model. The maximum clade credibility tree is colored according to clade origin as shown on the right, with B in orange, intermediate B0 and C0 in pink, C1 in light green, and C2 in dark green. The x axis indicates emergence time estimates of the corresponding strains. Node bars represent 95% highest posterior density intervals. Download [file mbo002162781sf9.pdf]
