# Supplementary material for: Sequential Acquisition of Virulence and Fluoroquinolone Resistance Has Shaped the Evolution of Escherichia coli ST131
Source: mBio. 2016 Apr 26;7(2):e00347-16. doi: 10.1128/mBio.00347-16 (PMC4850260; doi:10.1128/mBio.00347-16)
Supplement: Figure S7 — Prevalence of representative virulence factors in ST131 strains. Screening for the presence/absence of virulence factors was performed using the BLAST-based visualization tool SeqFindR (41). Virulence genes are shown along the x axis with strain identifiers on the y axis in the same order as the phylogenetic tree shown on Fig. S2B in the supplemental material. Virulence genes are listed in groups and correspond to (i) autotransporters, (ii) chaperone usher fimbriae, (iii) iron uptake, (iv) other virulence genes, (v) toxins, or (vi) UPEC-specific genes. Black shading shows a match of ≥95% nucleotide identity when comparing the query sequence to the assemblies or the consensus generated from read mapping. Download [file mbo002162781sf7.pdf]

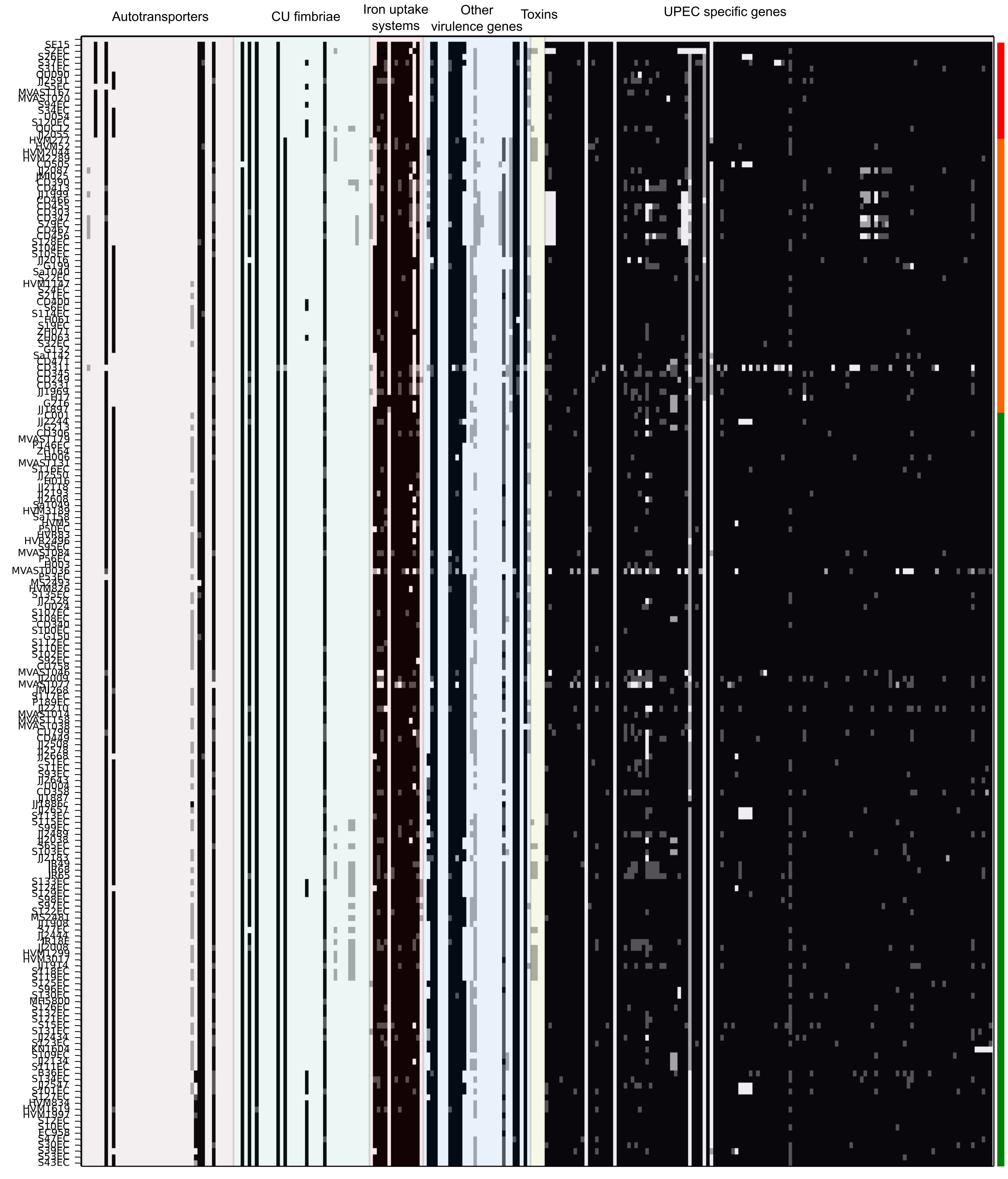
Autotransporters
CU fimbriae
Iron uptake systems
Other virulence genes
Toxins
UPEC specific genes
